# Pollen tube emergence is mediated by ovary-expressed ALCATRAZ in cucumber

**DOI:** 10.1038/s41467-023-35936-z

**Published:** 2023-01-17

**Authors:** Zhihua Cheng, Xiaofeng Liu, Shuangshuang Yan, Bin Liu, Yanting Zhong, Weiyuan Song, Jiacai Chen, Zhongyi Wang, Gen Che, Liu Liu, Ao Ying, Hanli Lv, Lijie Han, Min Li, Jianyu Zhao, Junqiang Xu, Zhengan Yang, Zhaoyang Zhou, Xiaolan Zhang

**Affiliations:** 1 State Key Laboratories of Agrobiotechnology, Joint International Research Laboratory of Crop Molecular Breeding, Beijing Key Laboratory of Growth and Developmental Regulation for Protected Vegetable Crops, Department of Vegetable Sciences, China Agricultural University, 100193 Beijing, China; 2 Centre for Research in Agricultural Genomics (CRAG), CSIC-IRTA-UAB-UB, Campus Universitat Autònoma de Barcelona, Bellaterra, 08193 Spain; 3 College of Horticulture and Landscape, Yunnan Agricultural University, 650201 Kunming, Yunnan China

**Keywords:** Fertilization, Plant molecular biology

## Abstract

Pollen tube guidance within female tissues of flowering plants can be divided into preovular guidance, ovular guidance and a connecting stage called pollen tube emergence. As yet, no female factor has been identified to positively regulate this transition process. In this study, we show that an ovary-expressed bHLH transcription factor *Cucumis sativus* ALCATRAZ (CsALC) functions in pollen tube emergence in cucumber. *CsALC* knockout mutants showed diminished pollen tube emergence, extremely reduced entry into ovules, and a 95% reduction in female fertility. Further examination showed two rapid alkalinization factors *CsRALF4* and *CsRALF19* were less expressed in *Csalc* ovaries compared to WT. Besides the loss of male fertility derived from precocious pollen tube rupture as in *Arabidopsis*, *Csralf4 Csralf19* double mutants exhibited a 60% decrease in female fertility due to reduced pollen tube distribution and decreased ovule targeting efficiency. In brief, CsALC regulates female fertility and promotes *CsRALF4/19* expression in the ovary during pollen tube guidance in cucumber.

## Abstract (web summary)

Pollen tube growth is guided towards ovules. Here the authors show that a bHLH transcriptional factor CsALC functions in pollen tube emergence towards ovules to regulate female fertility in cucumber and promotes the expression of two rapid alkalinization factors CsRALF4/19 in the ovary.

## Introduction

**Fig. 1:**
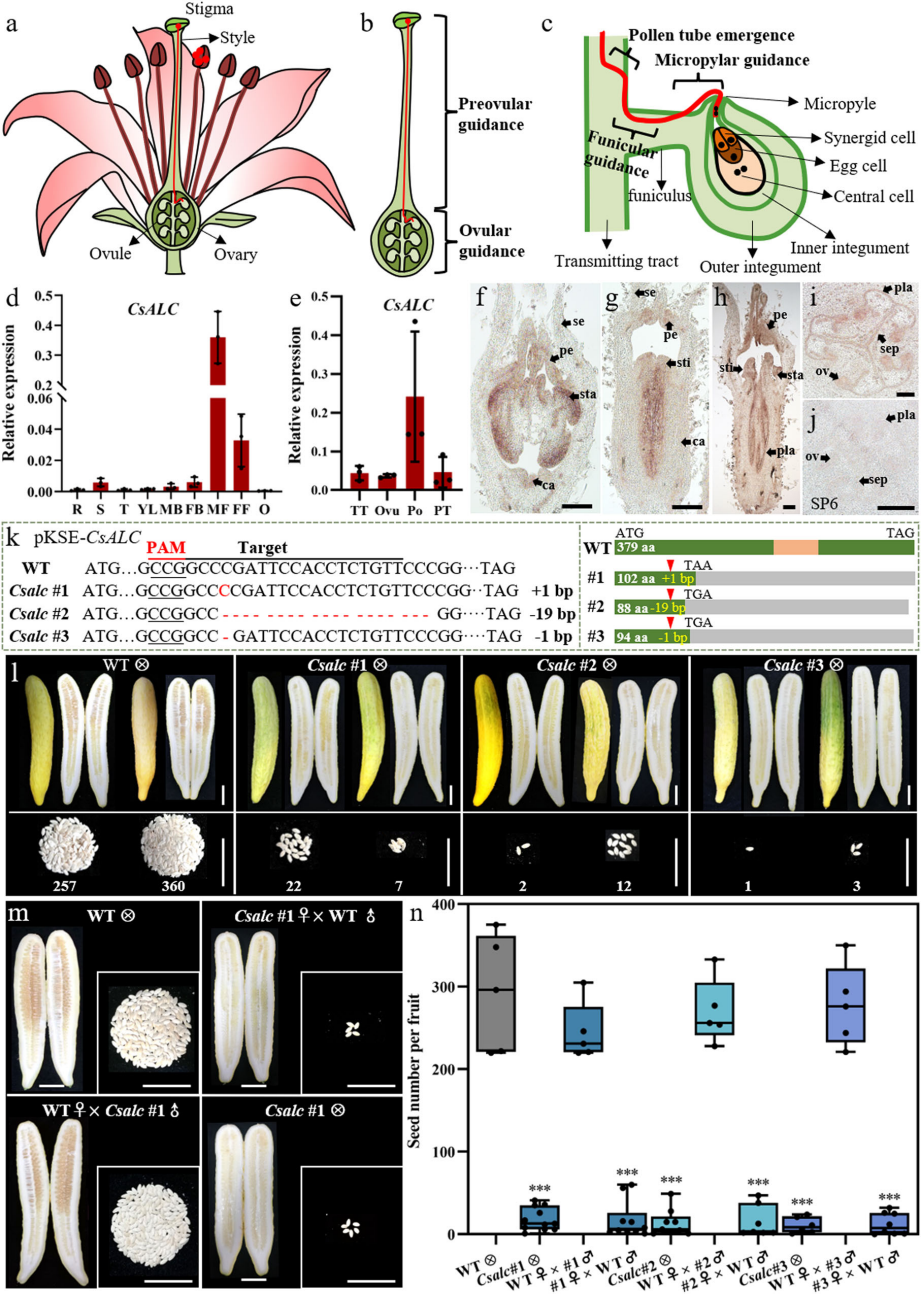
*CsALC* expression pattern in reproductive organs and defective female fertility of *Csalc* mutants by CRISPR-Cas9. **a–c** Schematic diagram of pollen tube guidance during the process of fertilization in flowering plants. **a** Schematic diagram of a flower showing the pollen tube penetrates through the stigma and style, enters the ovary, and targets an ovule. **b** Two phases of pollen tube guidance: preovular guidance and ovular guidance. **c** One pollen tube emerges from the transmitting tract (pollen tube emergence process) by ovule attraction, then extends towards the embryo sac under funicular guidance and micropylar guidance. **d**, **e** qPCR analysis of *CsALC* expression in different cucumber organs. R roots, S stems, T tendrils, YL young leaves, MB male flower buds, FB female flower buds, MF male flowers, FF female flowers, O ovaries at anthesis, TT ovary transmitting tract at anthesis, Ovu ovules at anthesis, Po pollens at anthesis, PT pollen tubes at 3 h after germination. The vertical axis refers to the relative *CsUBI* (CsaV3_5G031430) expression. *n* = 3 biologically independent samples. Error bars represent mean ± SD. **f**–**j** In situ hybridization of *CsALC* in cucumber flower buds. **f** A male flower bud at stage 9. **g**, **h** Longitudinal sections of female flower buds at stage 8-1 (**g**), and 8-2 (**h**). **i** Ovary transection of a female flower bud at stage 9. **j** Ovary transection of a female flower bud at stage 8 hybridized with sense probe of *CsALC* as the negative control. se sepal, pe petal, sta stamen, sti stigma, ca carpel, pla placenta, sep septum, ov ovule. Scale bars = 200 μm. The experiment was repeated three times with similar results. **k** Mutation forms of three homozygous T1 *Csalc* mutant lines by CRISPR-Cas9 system. The target sequence and PAM are marked by a black line and a red line, respectively. Deletions/ insertions are indicated as red dashes/ nucleotides, and corresponding nucleotide sizes are marked after the sequence as a minus or plus, respectively. The right dotted box shows the corresponding protein premature termination. The green/gray boxes indicate the protein-coding region and missense sequences resulting from frameshift mutations, respectively. The orange box in WT represents the bHLH conserved domain in CsALC. aa amino acid. **l** Seed number of wild type (WT) and *Csalc* mutant lines by self-pollination. Pictures represent the mature fruit, its longisection, and all plump seeds harvested from it, with the corresponding number at the bottom. Scale bars = 5 cm. **m** Reciprocal cross between *Csalc* #1 mutant and WT. Scale bars = 5 cm. The experiments were repeated three times with similar results (**l**, **m**). **n** Quantitative analysis of seed numbers from (**l**, **m**). Upmost/lowest lines indicate the maximum/minimum values, box limits indicate the upper and lower quartiles, and lines in boxes indicate the median values of these data. From left to right, *n* = 5, 11, 5, 10, 9, 5, 7, 6, 5, 8 mature fruits. ***p < 0.001, compared to WT (two-sided Student’s *t*-test).

Seed production is critically important for sexual propagation in agricultural crops. Successful delivery of sperm cells (contained within the pollen tube) to female gametes enclosed inside the ovary is a prerequisite for seed genesis^1–3^. After germinating on the stigma, pollen tubes extend and navigate along the female reproductive tract (transmitting tract: TT) towards ovules^1,4^ (Fig. 1a). During this process, the pollen tube perceives signal substances from both female sporophytic and gametophytic tissues of the pistil, which guide it towards the embryo sac, where the pollen tube penetrates into the synergid and releases two sperm cells, with one fusing with the egg cell and the other with the central cell^5,6^. Pollen tube guidance can be divided into preovular guidance (before entering into the ovary) and ovular guidance (Fig. 1b); the latter step is further divided into funicular guidance (guidance from the septum surface to the funiculus) and micropylar guidance (guidance from funiculus to micropyle and embryo sac) (Fig. 1c)^5,7,8^. Between preovular and ovular guidance, there is a crucial transition phase called pollen tube emergence, in which pollen tubes exit from the transmitting tract and move onto the septum surface (Fig. 1c).

Accurate pollen–pistil interactions occur during the long journey from stigma to ovules, functional disruption of either male gametophytes or female reproductive tissues will bring obstacles to oriented pollen tube growth, and thus lead to failure of double fertilization^9^. Upon landing on the stigma, *Arabidopsis* compatible pollen grains germinate via precise interactions between the pollen and pistil involving POLLEN COAT PROTEIN B-class peptides and ANJEA-FERONIA receptor kinase complex^10^. In tomato (*Lycopersicon esculentum*), LeSTIG1, a cysteine-rich protein secreted from the pistil, can bind to the pollen-specific receptor kinases LePRK1 and LePRK2 to stimulate pollen tube growth^11^. *Arabidopsis* pollen tube-specific cysteine-rich peptides (CRPs), Rapid Alkalinization Factor 4 (RALF4), and RALF19^12,13^, together with their pollen tube located *Catharanthus roseus* Receptor Like Kinase 1 Like (CrRLK1L) receptors BUDDA’S PAPER SEAL1 (BUPS1)/ BUPS2^12^, and ANXUR1 (ANX1)/ ANX2^14,15^, and co-receptors glycosylphosphatidylinositol-anchored proteins (GPI-APs), LORELEI-like-GPI-anchored protein 2 (LLG2) and LLG3^16^, are demonstrated to control pollen tube integrity and growth. Pollen tubes in double mutant *ralf4 ralf19*^12,13^, *anx1 anx2*^14,15^, *bups1 bups2*^12^, *llg2 llg3*^16^ all burst precociously at stigma or not beyond the style, resulting in extension cutoff at preovular stage.

“Ovular guidance” attracts one pollen tube to its target ovule, forming a specific one-to-one relationship^5^. Besides *Arabidopsis* MAGATAMA1 (MAA1: an unknown type protein) and MAA3 (a kind of helicase)^8,17^, several attractant peptides produced from female gametophytes have been identified to govern ovular guidance, including the Gramineae-specific small peptide EGG APPARATUS1 (EA1) in maize^18,19^, defensin-like CRPs LUREs and XIUQIUs in *Torenia fournieri*^20^ and *Arabidopsis*^21,22^. In *Arabidopsis*, the leucine-rich repeat receptor-like kinase (LRR-RLK) PRK6, localized at the pollen tip, was identified as the receptor for LURE1s^23^. Unlike that knock-down of *EA1* gene resulted in ovule sterility in maize, neither *Arabidopsis allure1* septuple mutants nor *prk6* mutant exhibited defects in fertility. Simultaneous knockout of both 7 LURE1s and 4 XIUQIUs led to only a 20% decrease in female fertility, indicating the redundant roles between LURE1s and XIUQIUs, and additional attractants in *Arabidopsis* micropylar guidance^22,24^.

Pollen tube emergence as the transition phase between preovular and ovular guidance refers to the first re-orientation of pollen tubes extending from the transmitting tract to the septum surface before proceeding onto funiculus, which was speculated to be affected by ovules to some extent^5^. In Arabidopsis, pollen tubes that lost the function of two endoplasmic reticulum-localized cation/proton exchangers CHX21 and CHX23 exhibited normal growth but were unable to emerge from the transmitting tract, showing a defect in pollen tube guidance^25^. Recently, pollen tube-expressed RALF6, 7, 16, 36, and 37 were shown to interact with female tissue-located receptor-like kinases FERONIA, ANJEA, and HERCULES RECEPTOR KINASE 1 (HERK1) to control the emergence of a single pollen tube at the septum for each ovule^26^. The multiple mutants of *RALFs* or *RLK* genes showed polytubey phenotype (arrival of multiple pollen tubes to one ovule)^26^. Although it was proposed there exists long-range attraction from ovules, no female sporophytic genes have been identified to positively regulate pollen tube emergence in *planta* so far^5^.

*ALCATRAZ* (*ALC*) encodes a bHLH transcription factor that mediates fruit opening by driving the formation of a separation layer at the valve/replum border that constitutes the dehiscence zone with the lignified layer in *Arabidopsis*^27^. Cucumber (*Cucumis sativus* L.) is an important vegetable crop bearing pepo fruit cultivated worldwide for over 3000 years^28^. In this study, we found cucumber ALC homolog (CsALC) functioned specifically in pollen tube emergence, regulated female fertility, and promoted TT-expressed *CsRALF4/19* during pollen tube growth in cucumber.

## Results

### *CsALC* is highly expressed in reproductive organs of cucumber

To identify putative fruit developmental regulators in the fleshy pepo fruit, the cucumber homolog of *Arabidopsis* bHLH transcription factor ALC, functioning in fruit opening, was searched in Cucurbit Genomics Database (CuGenDB: http://cucurbitgenomics.org/), and named as CsALC (Csa2G356640.1). ALC was reported to be the closest relative to SPATULA (SPT)^29^. Phylogenetic analysis showed there were SPT lineage and ALC lineage within core eudicots (Supplementary Fig. 1a). *CsALC* gene contains six exons and five introns, and its encoded protein is located in the nucleus, with 379 amino acids and a typical bHLH domain (Supplementary Fig. 1b–d). qPCR analysis showed that *CsALC* transcripts accumulate predominately in reproductive organs, including male flowers and female flowers (Fig. 1d). During fertilization, *CsALC* was expressed in transmitting tract (TT), ovules, pollens, and pollen tubes (Fig. 1e). In situ hybridization further localized *CsALC* transcripts in petals, stamens, and degenerated carpel primordia in male flower buds (Fig. 1f). In female flower buds, there was substantial enrichment of *CsALC* signals in developing carpels, especially placenta and stigmas, as well as in petals and degraded stamen primordia (Fig. 1g, h). Ovary transverse sections showed that compared to the negative control, prominent *CsALC* signals were localized in ovules, placenta, and septum (two parts where transmitting tracts are generated for fertilization in cucumber) at female flower bud stage (Fig. 1i, j).

### Knockout of *CsALC* resulted in reduced female fertility in cucumber

To gain insight into CsALC function in cucumber, the first exon of *CsALC* was targeted by CRISPR-Cas9 gene-editing system (Supplementary Fig. 1b). Three homozygous frameshift mutant lines were obtained for further characterization (Fig. 1k). Compared to the wild type (WT), *Csalc* mutant lines exhibited similar plant growth and fruit length (Supplementary Fig. 2a–c). However, the seed number per fruit in *Csalc* mutant lines was dramatically reduced after self-pollination (Fig. 1l), and the few seeds were randomly distributed in the fruit chamber of *Csalc* mutants. Considering the higher expression of *CsALC* in male gametophytes (Fig. 1e), we first investigated the pollen development and viability by Alexander staining and in vitro germination assays. As shown in Supplementary Fig. 2d, *Csalc* pollen grains were morphologically normal and able to germinate and grow on a medium. Quantitative analysis showed the slightly reduced viability of *Csalc* pollens (90.4 ± 1.6%, 90.6 ± 1.7%, 90.6 ± 1.1%, *p* < 0.001 versus WT 97.9 ± 1.4% by Alexander staining assay; 94.9 ± 1.3% *p* < 0.01, 94.9 ± 0.7% *p* < 0.001, 95.2 ± 1.8% *p* < 0.05 versus WT 99.2 ± 0.5% by in vitro germination assay) (Supplementary Fig. 2e, f). This mild decrease in pollen viability has a negligible impact on the cucumber seed set since an excess of pollens is usually available during open pollination.

Next, reciprocal crosses between *Csalc* mutants and WT were performed. As shown in Fig. 1m, n, when *Csalc* pollens were pollinated to WT, high seed set was acquired (244.6 ± 35.3, 269.6 ± 39.5, 277.0 ± 49.6 seeds per fruit), similar to that in WT self-crossing (292.5 ± 70.9). In contrast, when *Csalc* mutants were fertilized with WT pollens, a drastically lower seed set (17.2 ± 22.1, 15.3 ± 19.2, 12.5 ± 13.3, *p* < 0.001 versus WT self-crossing) was obtained, equivalent to those in *Csalc* mutant self-pollination (18.3 ± 13.9, 13.7 ± 15.9, 11.0 ± 9.6, *p* < 0.001 versus WT self-crossing), resulting in an average of 95% (respective 94.1%, 94.8%, 95.7%) decrease in seed number per fruit compared to WT. The above data suggested *Csalc* mutants have a remarkable defect in female fertility. When a heterozygous+/*Csalc* mutant self-crossing, the +/+, +/*Csalc*, *Csalc/Csalc* in the 213 progeny seedlings showed segregation of 53:117:43, approximating the ratio of 1:2:1, suggesting that the defect in *Csalc* mutant is localized to female sporophytic tissues.

### Pollen tubes displayed difficulty in targeting ovules in *Csalc* mutants

**Fig. 2:**
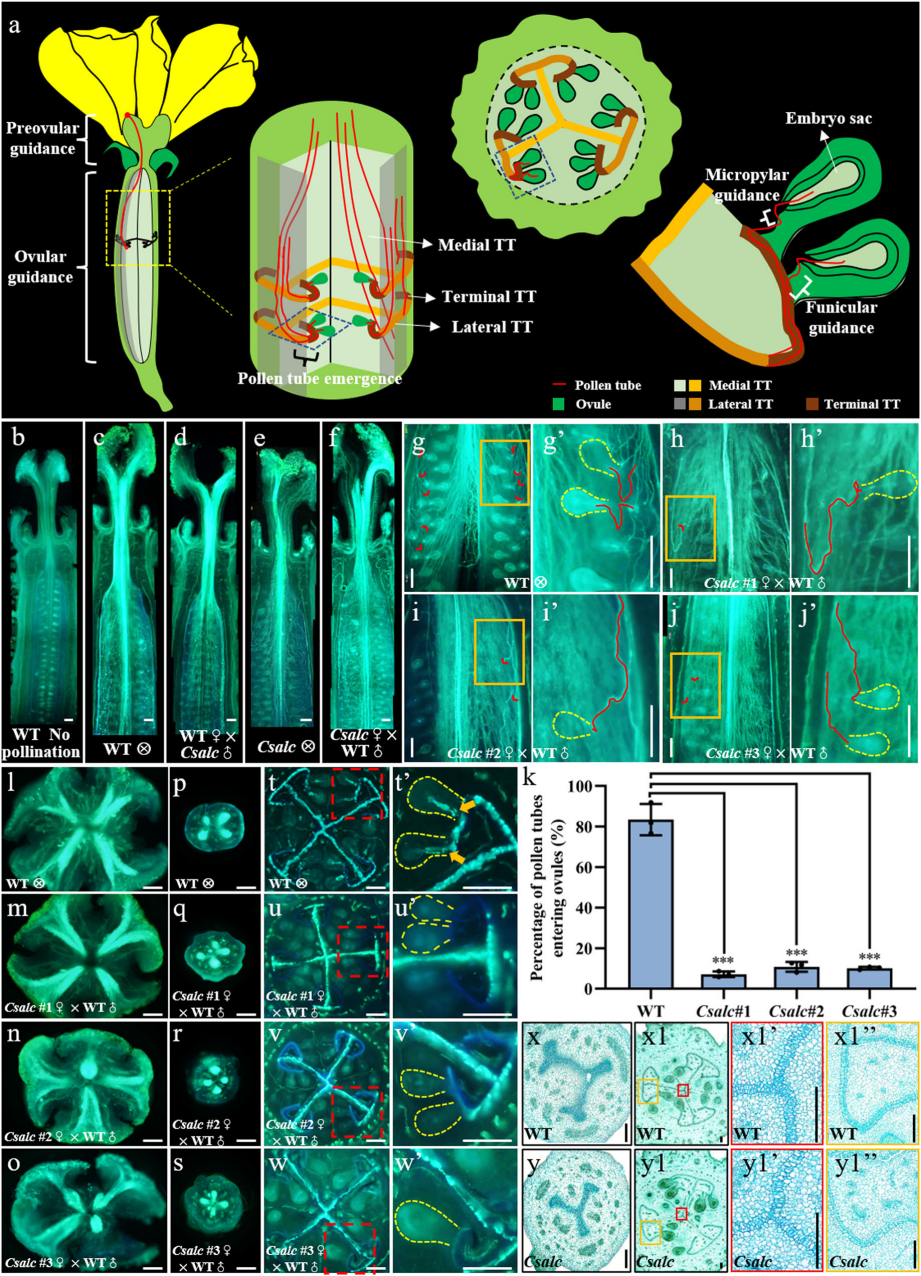
Pollen tubes are less likely to reach ovules in the *Csalc* ovary. **a** Schematic diagram of pollen tube paths along the female reproductive tract in cucumber, in which the ovary transmitting tract (TT) consists of medial TT [pale green (longitudinal section) and orange (transverse section)], lateral TT [gray (longitudinal section) and light brown (transverse section)] and terminal TT (dark brown). **b**–**f** Longitudinal sections showing pollen tubes extend along the transmitting tract from top to bottom in WT and *Csalc* ovaries. Composite images were integrated and spliced from local images taken separately. Scale bars = 500 μm. **g**–**j’** Typical images showing WT pollen tubes enter ovules in WT and *Csalc* mutants by longitudinal sections. Red arrow heads indicate pollen tubes targeting ovules. **g’**–**j’** Enlarged view of the orange boxes in (**g**–**j**). Targeted ovules are delineated by yellow dots, and pollen tubes are marked by red lines. Scale bars = 500 μm. **k** Quantitative analysis of pollen tubes entering ovules in WT and *Csalc* mutants. *n* = 3 biologically independent samples. Error bars represent mean ± SD. ***p < 0.001, compared to WT (two-sided Student’s *t* test). **l**–**w’** Transverse sections displaying the diminished distribution of pollen tubes at terminal transmitting tract in *Csalc* ovaries, and pollen tubes unable to target ovules. **l**–**o** stigma, **p**–**s** style, **t**–**w** the top quarter of the ovary. **t’**–**w’** Enlarged view of red dotted boxes in (**t**–**w**). The ovules are delineated by yellow dots. Orange arrows refer to pollen tubes entering ovules. Scale bars = 500 μm. **x**–**y1”** Cell morphology and structural composition of the transmitting tract in styles (**x**, **y**) and ovaries (**x1**, **y1**) of WT (top row) and *Csalc* mutant (bottom row) by Alcian blue staining. (**x1’**, **y1’**) and (**x1”**, **y1”**) are enlargement of red and orange boxes in (**x1**) and (**y1**), respectively. Scale bars = 200 μm.

Successful fertilization in flowering plants requires accurate pollen tube guidance in pistils, which can be divided into preovular guidance, ovular guidance, and a transition phase called pollen tube emergence^5^. As shown in Fig. 2a, we illustrated these processes in cucumber. Unlike the dehiscent silique with two cavities in *Arabidopsis*, cucumber produces fleshy fruit, which is generally developed from three dorsally fused carpels, and the ovules encased by flesh are born at both edges of each carpel. The distinct carpel fusion form in cucumber gives rise to the unique morphology of the transmitting tract (TT), which is divided into medial TT, lateral TT, and terminal TT in ovary^30^ (Fig. 2a). After entering into ovaries, pollen tube bundles firstly extend along medial TT to the nearby lateral TT, then after a short extension, pollen tubes exit from lateral TT to terminal TT, resembling *Arabidopsis* pollen tube emergence process in which pollen tubes start to depart from the main growth axis to target ovules (Fig. 2a). Therefore, this first re-orientation in cucumber is designated as “pollen tube emergence”. The second re-orientation from terminal TT to funiculus is depicted as funicular guidance (Fig. 2a). After extremely short navigation along the funiculus, a third re-orientation is likely triggered by micropylar guidance cues at a much closer range from micropyle (Fig. 2a).

To dissect the defective step of fertilization in *Csalc* mutants, Aniline blue staining was used to visualize pollen tube elongation and penetration in pistils. Compared to the WT ovary without pollination (Fig. 2b), obvious fluorescence signals showed the movement trail of pollen tubes from the stigma to style, ovary chamber, and finally the ovules in WT self-crossing or hybridized with *Csalc* pollens (Fig. 2c, d). In *Csalc* ovaries, pollen tubes spread normally downward along the medial transmitting tract and maintained similar fluorescence intensity with those in WT (Fig. 2e, f). By local longitudinal sections, abundant pollen tubes could be observed emerging from lateral TT and entering ovules in WT (Fig. 2g–g’), while rare in *Csalc* mutants (Fig. 2h–j’). Quantitative analysis showed that 83.5 ± 7.7% pollen tubes successfully targeted ovules in WT, while only 7.1 ± 1.4%, 10.8 ± 2.4%, 10.1 ± 0.7% in *Csalc* #1, #2, #3 mutants, respectively (Fig. 2k). Transverse sections of stigmas and styles showed comparable fluorescence intensity of pollen tubes at the stigma and style transmitting tract in *Csalc* mutants to that in WT (Fig. 2l–s). While, the fluorescence signals in *Csalc* mutants were almost abolished at the terminal TT where funiculus attached (Fig. 2t–w), and pollen tubes were barely observed approaching and penetrating into ovules as compared to WT (Fig. 2t’–w'). By a time-course observation (2, 5, 10, 20 h), we found WT pollen tubes grew in *Csalc* transmitting tract at a similar rate to those in WT (Supplementary Fig. 3a). However, pollen tubes at lateral TT extended almost downward, and rarely spread to terminal TT or turned growth direction to where ovules located in *Csalc* mutants (Supplementary Fig. 3b, c). Therefore, the decreased female fertility in *Csalc* mutants is from the defect in pollen tube emergence from lateral TT to terminal TT. No difference was observed in tissue structure and cell morphology between *Csalc* mutant and WT transmitting tract, while the extracellular matrix (ECM) content in ovary TT detected by Alcian blue staining, an indicator of acidic polysaccharides, displayed a reduction in *Csalc* mutants (Fig. 2x–y1”). ECM, containing various glycoproteins, has been proposed to mediate the attraction, nutrition, and stimulation of pollen tube growth in *Arabidopsis*^4^.

### *CsRALF4/19* transcripts declined at the transmitting tracts in *Csalc* mutants

**Fig. 3:**
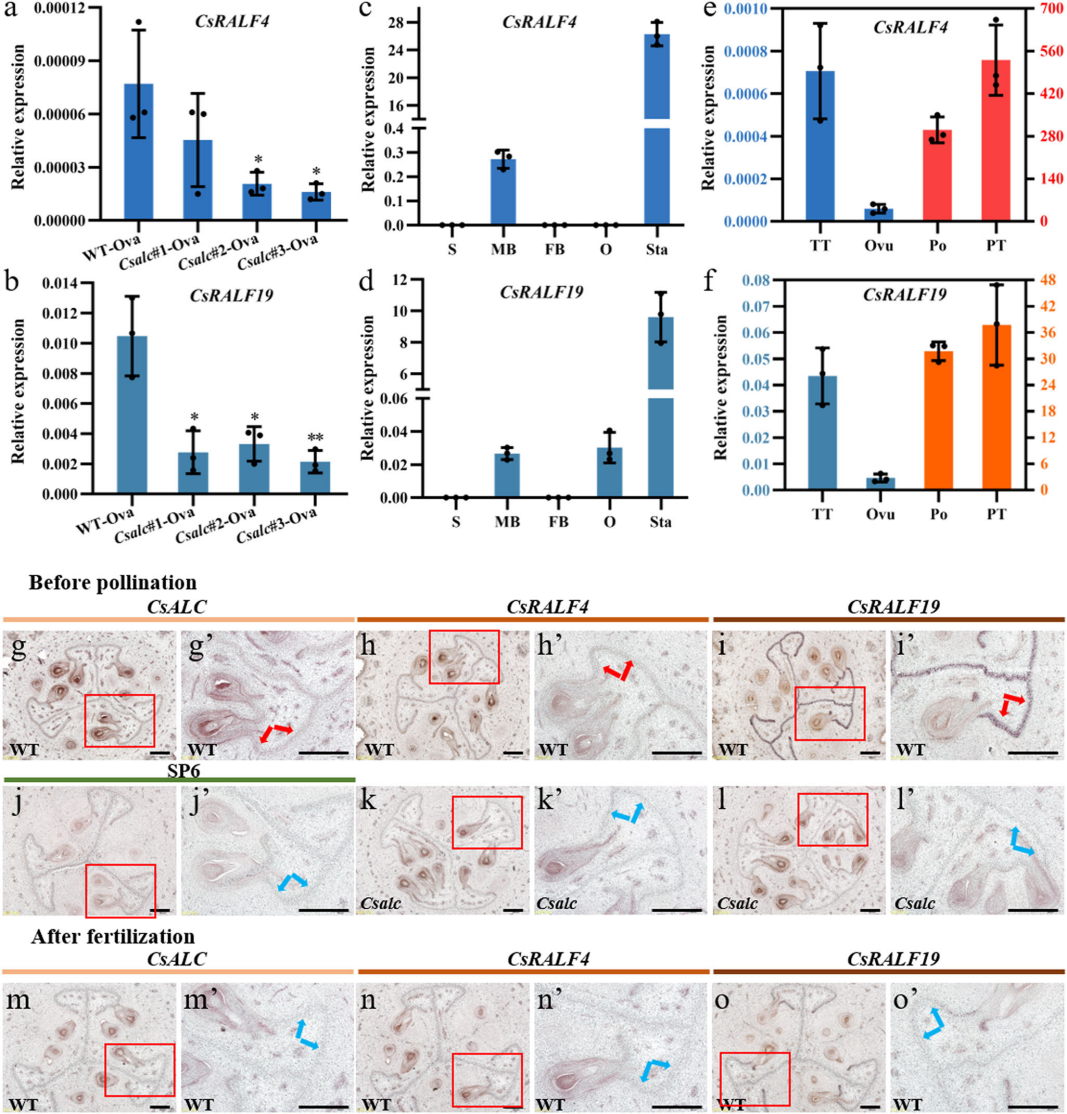
Reduced transcript accumulation of *CsRALF4* and *CsRALF19* in *Csalc* ovaries. **a**, **b** Expression of *CsRALF4* (**a**) and *CsRALF19* (**b**) in unpollinated ovaries at anthesis of WT and *Csalc* mutants by qPCR. *p < 0.05, **p < 0.01 (two-sided Student’s *t*-test). **c**, **d** *CsRALF4* (**c**) and *CsRALF19* (**d**) expression in different cucumber organs. S stems, MB male flower buds at stage 10^50^; FB female flower buds at stage 10^50^; O ovaries at anthesis; Sta stamens at anthesis. **e**, **f** *CsRALF4* (**e**) and *CsRALF19* (**f**) expression in female sporophytes and male gametophytes. TT ovary transmitting tract at anthesis, Ovu ovules at anthesis, Po pollens at anthesis, PT pollen tubes at 3 h after germination. The left/right vertical axis refers to the data of the blue/orange column, respectively, due to the magnitude difference of expression in different tissues. *n* = 3 biologically independent samples (**a–f**). The values were normalized with the *CsUBI* (CsaV3_5G031430) expression (**a-f**). Error bars represent mean ± SD (**a–f**). **g–o’** Transcript accumulation of *CsALC* (**g**, **g’**, **m**, **m’**), *CsRALF4* (**h**, **h’**, **k**, **k’**, **n**, **n’**), and *CsRALF19* (**i**, **i’**, **l**, **l’**, **o**, **o’**) at transmitting tract of unpollinated WT and *Csalc* ovaries (**g–l’**) and fertilized WT ovaries one day after anthesis (32 h after pollination) (**m–o’**) by in situ hybridization. **j**–**j’** SP6, Sense *CsALC* probe was hybridized as the negative control. **g’–o’** are enlargements of red boxes in (**g–o**). Red arrows indicate transcript signals at lateral- and terminal-TTs in WT before pollination, and blue arrows refer to the weakened transcript signals at lateral- and terminal-TTs in *Csalc* mutants, or in WT after fertilization. Scale bars = 500 μm. The experiment was repeated three times with similar results.

To explore putative downstream targets of CsALC during fertilization, transcriptomic analyses were performed on WT and *Csalc* ovaries at 32 h after pollination (Supplementary Fig. 4a). Differentially expressed genes (DEGs) were identified using the false discovery rate <0.05 and fold change larger than 2 as thresholds. A total of 8 and 20 genes were up- and down-regulated in *Csalc* ovaries, respectively (Supplementary Fig. 4b, c). Among them, a gene encoding rapid alkalinization factor (Csa6G484570, *CsRALF19*) was remarkably decreased (log2 fold change = 3.46, *P* = 3.49E−04) in *Csalc* mutants (Supplementary Fig. 4d). qPCR analysis using unpollinated ovary at anthesis showed that *CsRALF19* and its paralogous gene *CsRALF4* (Csa6G199790) were expressed significantly lower in *Csalc* mutants than WT (Fig. 3a, b). Phylogenetic analysis indicated that the two peptides clustered with *Arabidopsis* RALF4/19, which function in pollen tube integrity (Supplementary Fig. 5a)^12,13^. Unlike the pollen-specific expression of *Arabidopsis RALF4/19*, *CsRALF4/19* also have a certain degree of expression in the ovary, indicating their putative new function in female tissues in cucumber. *CsRALF4* and *CsRALF19* both contain a single exon and encode a 116- and a 112-amino-acid-long peptide, respectively (Supplementary Fig. 5b).

qPCR analysis showed that *CsRALF4*/*19* were highly expressed in mature stamens (Fig. 3c, d). However, unlike the differential expression of *CsRALF4*/*19* in female tissues of *Csalc* mutants and WT, the expression of these two genes in stamens was unchanged between *Csalc* mutants and WT (Supplementary Fig. 6a, b). Tissue subdivision showed that *CsRALF4/19* transcripts were enriched in pollen grains and pollen tubes. Notably, the expression of *CsRALF4/19* in the transmitting tract was higher than that in ovules (Fig. 3e, f). To compare the expression pattern in WT and *Csalc* mutants, in situ hybridization of *CsRALF4*/*19* and *CsALC* was performed on the transections of ovaries before pollination and after fertilization. In WT unpollinated ovaries, *CsRALF4*/*19* and *CsALC* transcripts were enriched at transmitting tract and ovules (Fig. 3g–i’), and *CsRALF19* exhibited much higher expression than *CsRALF4*, consistent with qPCR results (Fig. 3e, f). In *Csalc* unpollinated ovaries, *CsRALF4/19* transcript signals seemed weaker at TTs compared to WT (Fig. 3k, l’). The TT identity and morphology appeared unaltered, and the TT marker gene *SPATULA* (*CsSPT*) was expressed normally in *Csalc* ovaries compared to WT^30^ (Supplementary Fig. 6c). Considering the almost abolished pollen tube extension to terminal TT in *Csalc* ovaries (Fig. 2g–w), we speculated CsALC may regulate pollen tube emergence at least partially through *CsRALF4/19* function in cucumber. While after fertilization, transcripts of all three genes showed reduced accumulation (Fig. 3m–o’), indicating the potential specific roles of these genes in ovary TTs during the fertilization process.

### Positive regulation of CsALC on *CsRALF4/19* expression

To examine any direct regulation of CsALC on *CsRALF4/19* expression, the *cis-elements* in *CsRALF4/19* genes were analyzed. The bHLH transcription factor was shown to bind to the G-box of target genes^31^. G-box elements in the promoter of 28 DEGs between *Csalc* mutants and WT were searched on the PlantCARE website (http://bioinformatics.psb.ugent.be/webtools/plantcare/html/) (Supplementary Data 4). *CsRALF4* has one G-boxes in the promoter region, and one in the genomic region, and *CsRALF19* has two G-box in the promoter region, and one in the genomic region (Supplementary Fig. 7a). Yeast one hybrid (Y1H) assay showed that CsALC was unable to bind to any G-box containing fragments in *CsRALF4* and *CsRALF19* (Supplementary Fig. 7b). However, using the complete promoter sequence (1811 bp for *CsRALF4* and 3326 bp for *CsRALF19*), both *pCsRALF4:LUC* and *pCsRALF19:LUC* could be activated upon co-infiltration of CsALC protein in *Nicotiana benthamiana* leaves, increasing to 2.03 and 1.66 fold, respectively (Supplementary Fig. 7c). These results indicated the positive regulation of CsALC on *CsRALF4*/*19* expression in cucumber, consistent with the downregulation of *CsRALF4*/*19* transcripts in *Csalc* mutants as detected by qRT-PCR and in situ hybridization (Fig. 3).

### Knockout of both *CsRALF4* and *CsRALF19* led to reduced female fertility and a complete loss of male fertility

**Fig. 4:**
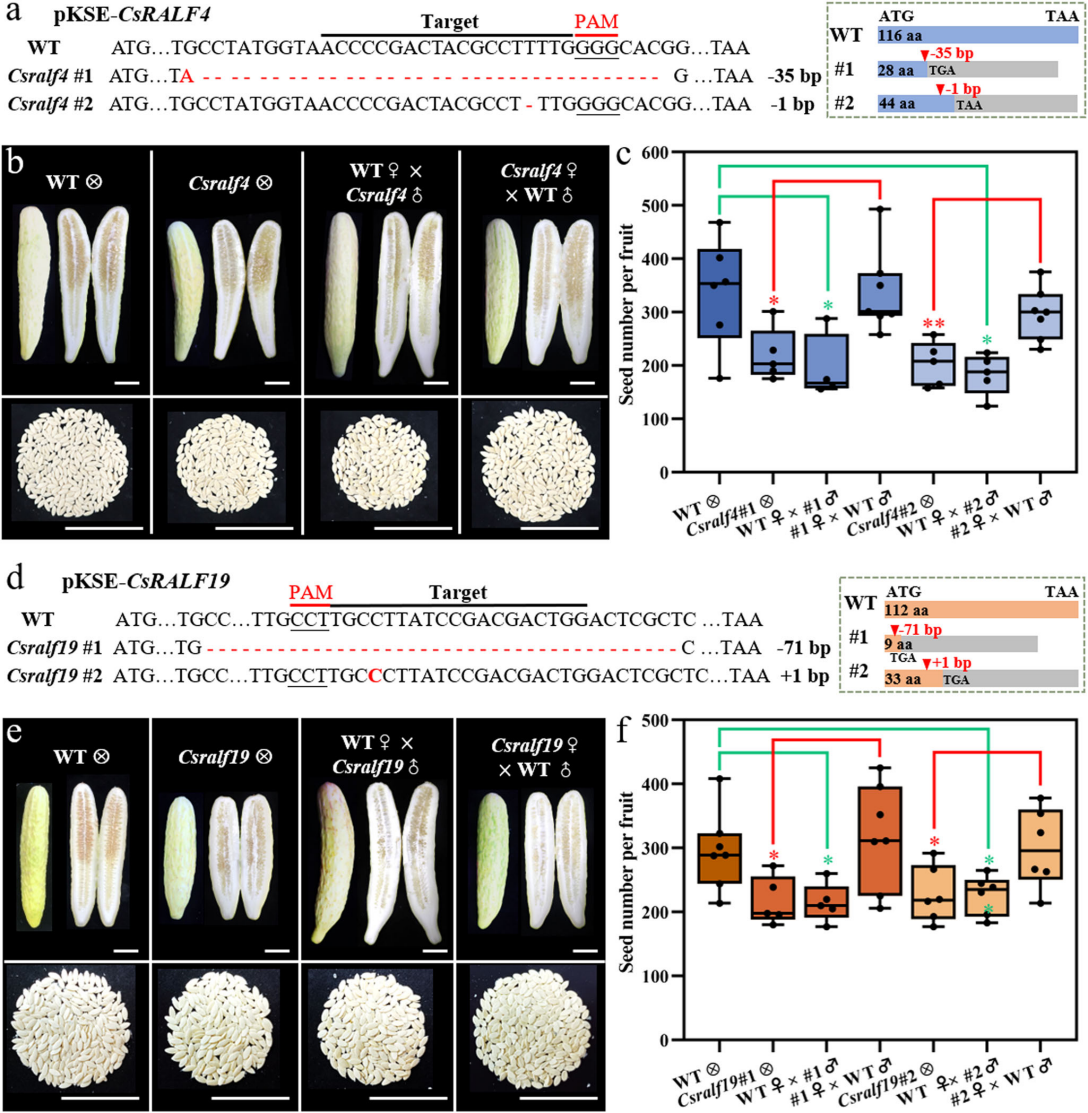
Decreased seed set in *Csralf4* and *Csralf19* single mutants. **a**, **d** Mutation forms of two homozygous T1 *Csralf4* lines (**a**) and two homozygous T1 *Csralf19* lines (**d**). Target sequences and PAMs are indicated below the black and red lines, respectively. Deletions/insertions are indicated as red dashes/nucleotides, and deletion/insertion sizes (nucleotides) are marked after the sequence as a minus or plus, respectively. The right dotted box shows the corresponding protein premature termination. The blue/orange boxes indicate CsRALF4/CsRALF19 protein coding region, respectively, and the gray indicates missense sequences resulting from frameshift mutations. aa amino acid. **b**, **e** Mature fruits, corresponding longisection, and harvested seeds from reciprocal crosses between *Csralf4* mutants (**b**)/*Csralf19* mutants (**e**) and WT. Scale bars = 5 cm. **c**, **f** Quantitative analysis of seed number from (**b**, **e**). Upmost/lowest lines indicate the maximum/minimum values, box limits indicate the upper and lower quartiles, and lines in boxes indicate the median values of these data. From left to right, *n* = 6, 5, 4, 7, 5, 5, 7 mature fruits in (**c**) and 7, 5, 5, 7, 6, 6, 6 mature fruits in (**f**), respectively. *p < 0.05, **p < 0.01 (two-sided Student’s *t*-test).

**Fig. 5:**
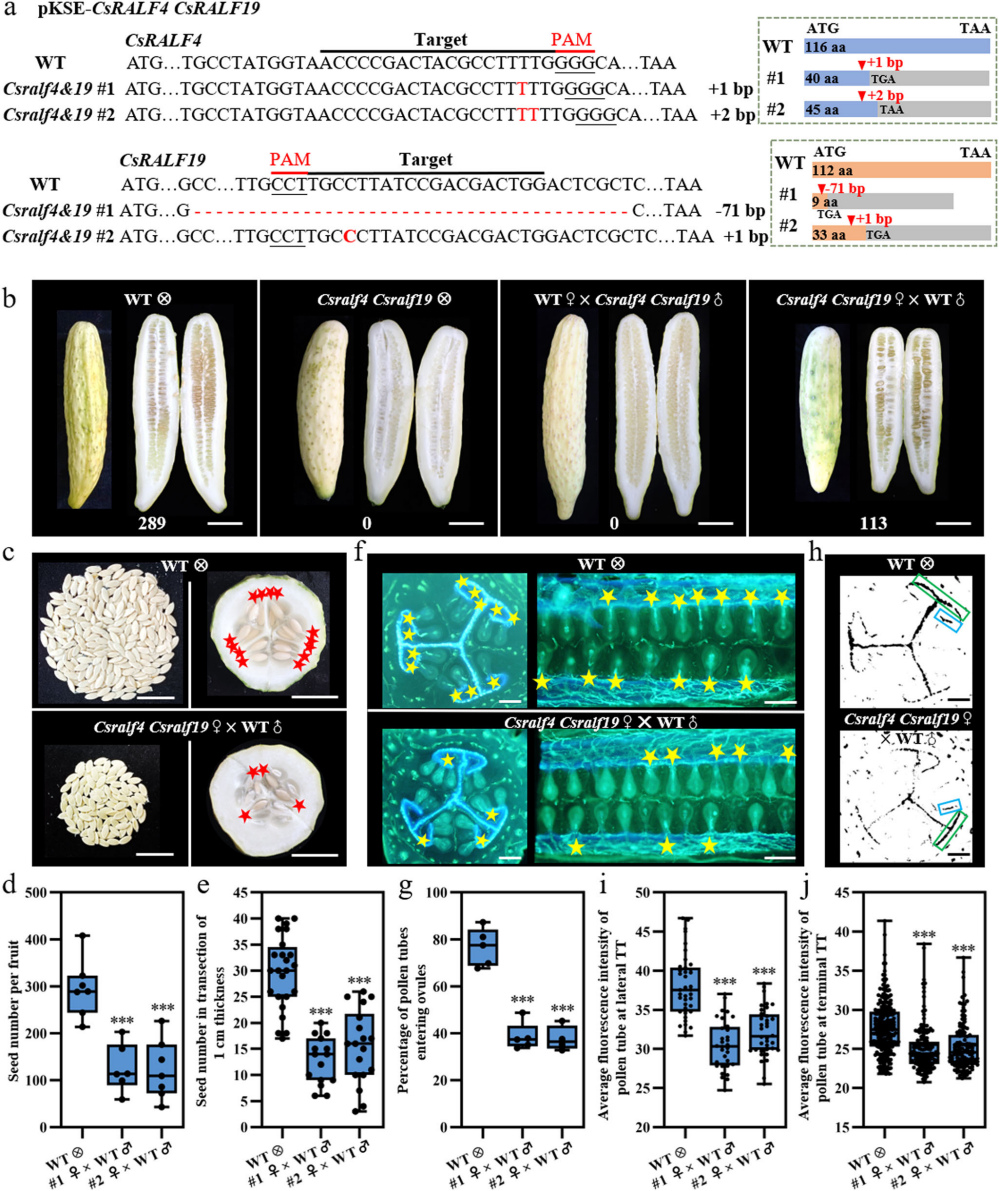
Reduced female fertility in *Csralf4 Csralf19* double mutants. **a** Mutation forms of two homozygous T1 transgenic *Csralf4 Csralf19* lines. The target sequence and PAM are indicated below a black and a red line, respectively. Deletions/insertions are indicated as red dashes/nucleotides, and deletion/insertion sizes (nucleotides) are marked after sequencing as a minus or plus, respectively. The right dotted box shows the corresponding protein premature termination. The blue/orange boxes indicate CsRALF4/CsRALF19 protein coding region, respectively, and the gray indicates missense sequences resulting from frameshift mutations. aa, amino acid. **b** Mature fruits and corresponding longisection from reciprocal crosses between *Csralf4 Csralf19* mutants and WT. The corresponding seed numbers were shown at the bottom. Scale bars =  5 cm. **c** All plump seeds and fruit transections from WT self-crossing and *Csralf4 Csralf19* hybridizing with WT pollens. Scale bars = 2 cm. **d**, **e** Quantitative analysis of seed number. From left to right, *n* = 7, 6, 7 mature fruits in (**d**) and 8, 5, 6 mature fruits in (**e**), respectively. **f** Aniline blue staining showing the ovule targeting in WT and *Csralf4 Csralf19* mutant ovaries at 32 h after pollination with WT pollens by transverse (left) and longitudinal sections (right). Yellow stars indicate ovules targeted by pollen tubes. Scale bars = 500 μm. **g** Quantitative analysis of pollen tubes entering ovules in (**f**). *n* = 5, 5, 4 biologically independent samples. **h** Pollen tubes distribution at lateral- and terminal-TT (pointed by green boxes and blue boxes, respectively) in ovary transections from (**f**). Scale bars = 500 μm. **I**, **j** Quantitative analysis of pollen tube fluorescence intensity at lateral- (**i**) and terminal-TT (**j**) in (**h**). The fluorescence threshold is 20–255. *n* = 39, 32, 36 lateral TTs in (**i**), and 235, 141, 120 terminal TTs in (**j**), respectively. For these Box-plots, upmost/lowest lines indicate the maximum/minimum values, box limits indicate the upper and lower quartiles, and lines in boxes indicate the median values of these data. ***p < 0.001 (two-sided Student’s *t*-test).

To characterize the function of CsRALF4/19 in cucumber, single mutants *Csralf4* (obtained by targeting the *CsRALF4* gene) and *Csralf19* (separated from *Csralf4 Csralf19* double mutants), as well as double mutant *Csralf4 Csralf19* (generated by simultaneously targeting *CsRALF4* and *CsRALF19*), were obtained by CRISPR-Cas9 system. Two homozygous lines for each mutant type were chosen for further characterization. Sequencing data showed that all mutant lines possessed frameshift mutations that resulted in protein premature termination (Figs. 4a, d, and 5a). Both *Csralf4* and *Csralf19* single mutants showed a reduced seed set when self-crossing (seed number per fruit: *Csralf4* mutants 219.6 ± 49.6, 203.0 ± 42.0, *p* < 0.05 versus WT 338.2 ± 101.6; *Csralf19* mutants 217.0 ± 37.7, 227.7 ± 43.9, *p* < 0.05 versus WT 295.4 ± 61.7). When *Csralf4* or *Csralf19* mutant ovaries pollinated by WT pollens, seed numbers (*Csralf4* mutants as maternal parents: 337.9 ± 78.4 and 296.9 ± 49.0; *Csralf19* mutants as maternal parents: 317.9 ± 81.6 and 300.0 ± 62.3) were comparable to WT self-crossing, suggesting the female fertility in both single mutants is normal. However, when *Csralf4/ Csralf19* mutants as pollen donors to WT ovaries, the resultant seed set was significantly reduced (194.5 ± 62.8, 183.2 ± 38.5, *p* < 0.05 for *Csralf4* mutants, and 214.4 ± 30.1, 226.5 ± 31.1, *p* < 0.05 for *Csralf19* mutants) versus WT self-crossing, suggesting the defective male fertility in *Csralf4* and *Csralf19* single mutants (Fig. 4b, c, e, f). Further, *Csralf4 Csralf19* double mutants completely lost male fertility and none seed was produced when as pollen donors (Fig. 5b). Intriguingly, when *Csralf4 Csralf19* double mutants hybridized with WT pollens, the seed number per fruit (125 ± 51.3, 122.3 ± 63.8, *p* < 0.001) was significantly fewer than WT self-pollination (295.4 ± 61.7), accounting for ~60% decrease in female fertility (Fig. 5c, d). The distribution of full seeds in the ovary chamber of *Csralf4 Csralf19* double mutants was scattered, similar to that in *Csalc* mutants. The average seed number per unit fruit length (1 cm) in *Csralf4 Csralf19* double mutants was 12.7 ± 4.5 (*p* < 0.001) and 15.5 ± 7.1 (*p* < 0.001), significantly reduced as compared to WT (29.6 ± 7.0) (Fig. 5c, e). Moreover, only 38.5 ± 5.9% (*p* < 0.001) and 37.7 ± 5.4% (*p* < 0.001) ovules were observed to be targeted by pollen tubes in two *Csralf4 Csralf19* double mutant lines, while the targeting ratio was 76.7 ± 8.1% in WT (Fig. 5f, g). The pollen tube density at the lateral- and terminal-transmitting tract in *Csralf4 Csralf19* ovaries was significantly reduced versus WT (Fig. 5h–j). These data suggested a new role of *CsRALF4*/*19* in female fertility in cucumber.

**Fig. 6:**
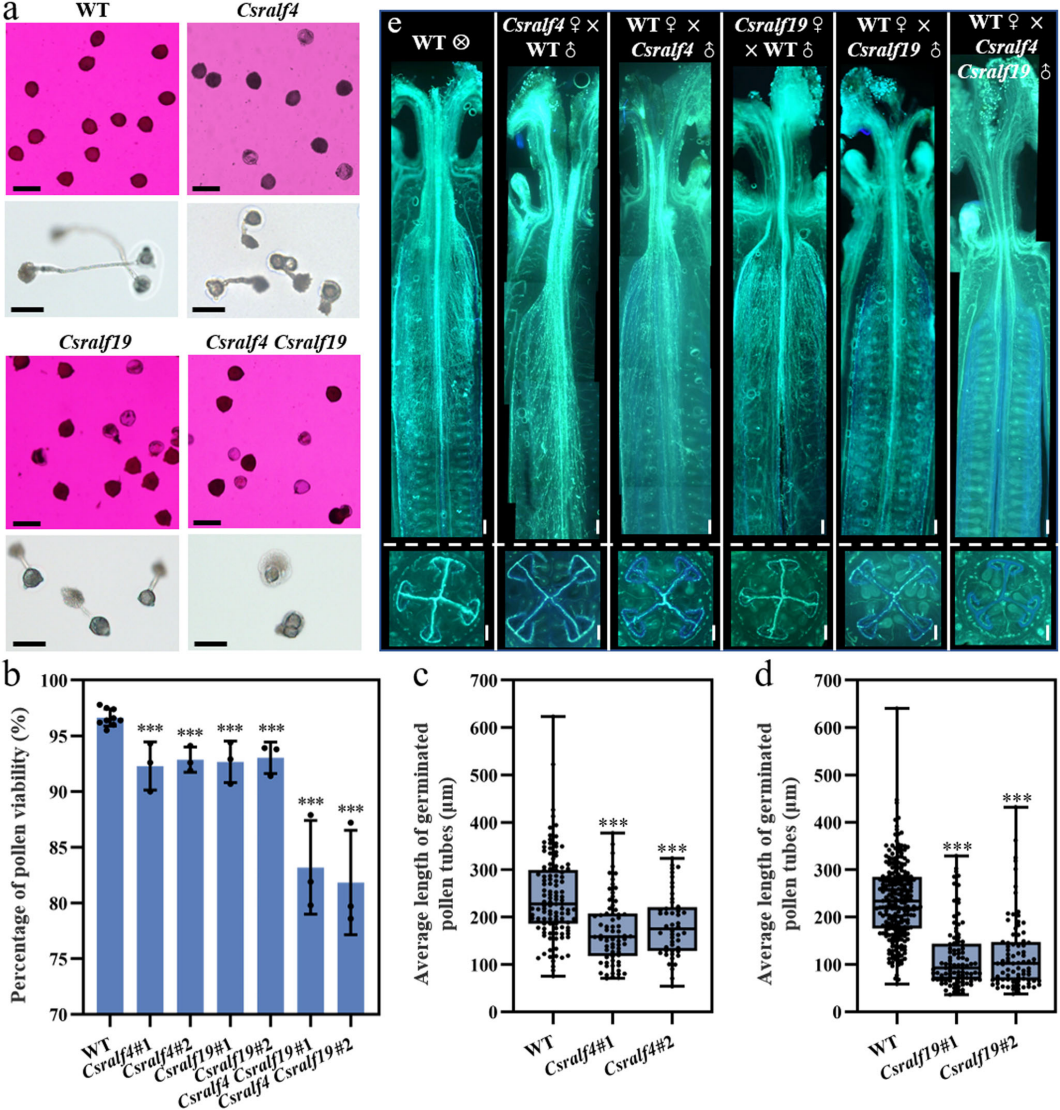
Abolished male fertility in *Csralf4 Csralf19* double mutants. **a** Alexander staining of pollens (top row) and in vitro pollen germination assay (bottom row) of *Csralf4*, *Csralf19* and *Csralf4 Csralf19* mutants. Scale bars = 100 μm. **b–d** Quantitative analysis of pollen viability (**b**) and pollen tubes length (**c**, **d**) from (**a**). *n* = 9, 3, 3, 3, 3, 3, 3 biologically independent samples in (**b**). Error bars represent mean ± SD. n = 115, 71, 48 pollen tubes in (**c**) and 240, 97, 83 pollen tubes in (**d**), respectively. ***p < 0.001 (two-sided Student’s *t* test). For these Box-plots, upmost/lowest lines indicate the maximum/minimum values, box limits indicate the upper and lower quartiles, and lines in boxes indicate the median values of these data. **e** Aniline blue staining showing the extension of pollen tubes by longitudinal (up) and transverse sections (bottom) in *Csralf4*, *Csralf19,* and *Csralf4 Csralf19* mutants. Composite images were integrated and spliced from local images taken separately. Scale bars = 500 μm.

Besides, defects in male gametophytes were detected in *Csralf4, Csralf19* single mutants, and *Csralf4 Csralf19* double mutants. Pollen viability was significantly reduced in all mutants (*Csralf4*: 92.3 ± 2.2%, 92.9 ± 1.1%; *Csralf19*: 92.7 ± 1.9%, 93.0 ± 1.4%; *Csralf4 Csralf19*: 83.2 ± 4.3%, 81.8 ± 4.7%; all *p* < 0.001) as compared to WT (96.6 ± 0.8%) (Fig. 6a, b). WT pollen tubes ruptured after reaching 242.9 ± 89.3 μm length, while pollens tubes in two *Csralf4* lines ruptured precociously at length of 172.0 ± 71.6 μm and 183.6 ± 67.1 μm (*p* < 0.001, Fig. 6a, c). Similarly, *Csralf19* pollen tube length was 115.2 ± 67.2 μm (#1) and 122.0 ± 77.3 μm (#2) versus WT 233.0 ± 80.2 μm (*p* < 0.001, Fig. 6a, d). In addition, the weakened fluorescence intensity by Aniline blue staining indicated poor extension of *Csralf4* and *Csralf19* pollen tubes in WT ovaries, especially at lateral- and terminal-TT (Fig. 6e). In *Csralf4 Csralf19* double mutants, pollen tubes burst immediately upon germination in vitro and on the stigmatic surface, which resembles the *Arabidopsis ralf4 ralf19* mutant phenotypes (Fig. 6a, e), suggesting the conserved function of CsRALF4/19 in pollen tube integrity^32^.

### *CsALC* was selected during cucumber domestication

Our findings showed the important function of CsALC in cucumber seed production and sustainable propagation. During cucumber domestication, the fruit is elongated while seed number per unit fruit length is generally decreased^33^. To explore any selection footprint on *CsALC*, the genomic region of *CsALC* plus its 3 kb promoter sequences (Chr02: 16738402–16744826) was analyzed in the published 115 cucumber accessions consisting of five populations: Indian wild genotypes (INW), Indian domestic (IND), Xishuangbanna (XSBN), East Asian (EA) and Eurasian (EU)^28^. The results showed that *CsALC* exhibited sharply reduced nucleotide diversity (π) in modern varieties (EA and EU), as compared to wild (INW) or semi-wild cucumber (XSBN) (Supplementary Fig. 8a). We also calculated the pairwise difference in allele frequency (FST) between domesticated groups and wild population INW, the results showed that *CsALC* was selected in Xishuangbanna, East Asian and Eurasian groups (Supplementary Fig. 8b), implying the importance of *CsALC* in cucumber domestication and breeding.

## Discussion

The ovary encloses and nourishes ovules, and ample evidence suggested that the ovary must provide an environment that supports pollen tube growth and guidance to the ovule^34^. Some female molecules, e.g. Ca^2+^ ions^35^, γ aminobutyric acid (GABA)^36,37^, brassinosteroids^38^, carbohydrates^4^, and various peptides^39^, have been characterized to stimulate pollen germination and pollen tube growth in preovular guidance process. These early stages are generally regulated by sporophytic genes such as those in the transmitting tract, e.g., *Transmitting-Tissue-Specific Arabinogalactan* gene (*TTS*) in tobacco^40^, *SPT*^29^, *HECATEs* (*HEC1/2/3*)^41^, *HALF FILLED* (*HAF*)^42^ in *Arabidopsis* and *CsSPT* in cucumber^30^. In *Csspt* mutants, the dramatically decreased polysaccharide at the style transmitting tract led to much less pollen tubes penetrating into ovaries and thus reduced seed set and female fertility^30^. Unlike that stimulant molecules promote the movement of pollen tubes at stigma or styles at preovular guidance stage, the attractant cues secreted from ovules generally trigger the re-orientation of pollen tube during the ovular guidance process (late stage of pollen tube guidance), e.g. the roles of CRP peptide LUREs and synergid cells during short range attraction^6,20,43^. Meanwhile, male factors such as receptor PRK6 and ion transporters CHX21/23 mediate pollen tube responsive behavior and competence for ovular attractions^23,25^. However, none female sporophytic gene has been reported yet to positively regulate pollen tube re-orientation at the transition stage between preovular guidance and ovular guidance, namely the pollen tube emergence process.

**Fig. 7:**
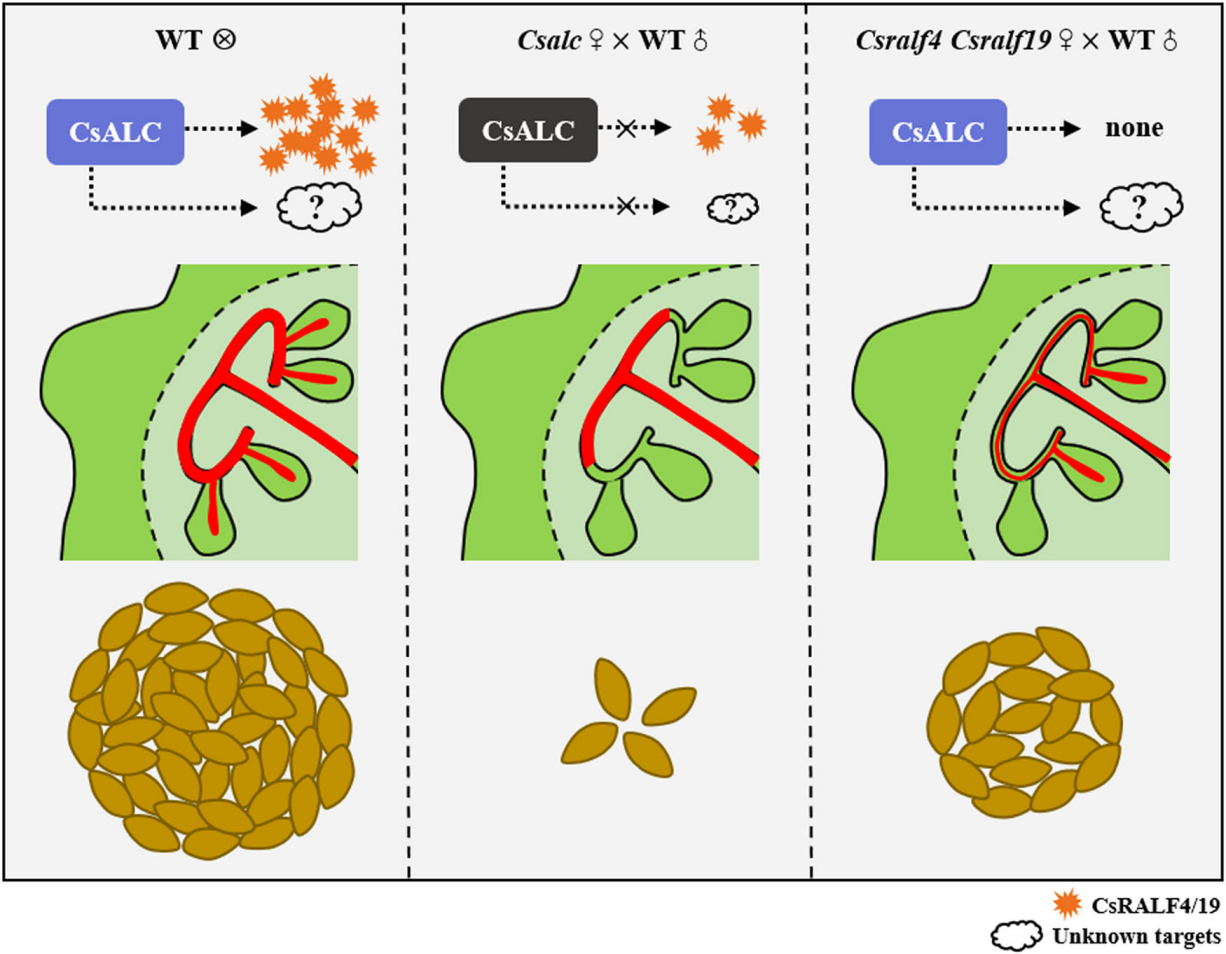
A working model for CsALC and CsRALF4/19 in pollen tube guidance in cucumber. In WT ovary, pollen tubes (in red) extend successively along the medial-, lateral- and terminal-TT and eventually enter ovules, producing hundreds of seeds per fruit in cucumber. In *Csalc* mutant, pollen tubes fail to re-orient to ovules and are blocked at the lateral TT, partially due to the decrease of CsRALF4/19 peptides, resulting in an approximately 95% reduction in seed number per fruit. In *Csralf4 Csralf1*9 mutant, the reduced pollen tube extension and decreased ovule targeting efficiency leads to about a 60% reduction of seed number in fruit. The much fewer seeds in *Csalc* mutants than in *Csralf4 Csralf1*9 mutants indicate there must be other downstream target genes.

In this study, we identified a transmitting tract-expressed gene *CsALC* that participates in pollen tube emergence in cucumber. Loss-of-function *Csalc* mutants displayed almost absence of pollen tube distribution at terminal TT and thus nearly 95% reduction in female fertility (Fig. 1). The movement of pollen tubes from top to bottom within *Csalc* pistils appeared normal while extending pollen tubes within *Csalc* ovaries failed to turn towards ovules, showing a defect in pollen tube emergence from lateral-TT to terminal-TT (Figs. 2 and 7). Unlike the hollow pod in *Arabidopsis*, cucumber bears the fleshy fruit, in which the cell–cell communication of pollen–pistil interactions mainly occurs at the transmitting tracts. From the physiological and molecular evidence, we can speculate that the transmitting tract circumstance in *Csalc* mutants is altered, in which main component polysaccharides and two TT-expressed peptides *CsRALF4* and *CsRALF19* were reduced (Figs. 2 and 3). The changed TT circumstance may affect the diffusion or distribution of some unknown ovular attractant molecules at lateral and terminal TT in cucumber, causing unresponsive pollen tube behavior. Another possibility is that the absence of female-assisted molecules at TTs, like phytohormones, peptides, and glycoproteins, may blunt the pollen tube perception for ovular signals or affect the directional growth of pollen tubes. The successful guidance for pollen tubes not only requires the secreted attractants but also the competency control from female tissues for pollen tube behavior^34^. It was reported that an ovary-abundant chemical cue, methyl-glucuronosyl arabinogalactan (AMOR), can promote pollen tube competence to respond to ovular attractant LURE peptides in *Torenia fournieri*^34^. The third possibility is that the mutant TT in *Csalc* may modify pollen tube status to lose the directional growth capacity. It may be operated through pollen-specific unknown receptors, or those genes involved in localized vesicle traffic, actin dynamics, or ion balance (e. g. *Arabidopsis* CHX21 and CHX23 in K^+^ homeostasis) that specify pollen tube tip growth and re-orientation^44^.

However, despite a similarly reduced extracellular matrix in *Csspt* ovaries, no pollen tube emergence defect was observed (Supplementary Fig. 9)^30^, suggesting the polysaccharides in TT may not be the critical factor required for proper pollen tube re-orientation. *CsSPT* mainly functions in the development of style TT that facilitates pollen tube extension from the stigma to the ovary. Mutation of *CsSPT* resulted in only upper portion ovules fertilized and thus a 60% reduction in seed set^30^. Considering *Csspt Csalc* double mutants displayed a complete loss of female fertility derived from the absence of TT, more severe than either *Csspt* or *Csalc* single mutants^30^, therefore, we conclude that CsALC and CsSPT have divergent functions in pollen tube guidance, while redundant roles in TT development in cucumber.

Besides the extremely high enrichment in pollens, *CsRALF4/19* were also expressed in female reproductive tissues in cucumber (Fig. 3). Knockout of both *CsRALF4* and *CsRALF19* resulted in pollen tube rupture prematurely and reduced female fertility from diminished pollen tube distribution at lateral and terminal-TT, and decreased ovule targeting efficiency (Fig. 5). These data suggested that in addition to the conserved function in pollen tube integrity by pollen-located CsRALF4/19 peptides^12,13^, the transmitting tract-expressed CsRALF4/19 peptides mediate pollen tube growth and extension in the cucumber ovary, probably by interaction with pollen tube-expressed RALF receptors similar to those in pollen tube integrity control^12^ (Fig. 7). Expression analysis indicated the CRPs *CsRALF4*/*19* transcripts were decreased in *Csalc* ovaries (Fig. 3). Although no direct interaction was found by yeast one hybrid, in vivo assay of *N. benthamiana* showed the positive regulation of CsALC on *CsRALF4/19* expression, perhaps with the assistance of other cofactors (Supplementary Fig. 7). Interestingly, the expression of *CsRALF4/19* was unchanged in the stamen of *Csalc* mutant (Supplementary Fig. 6), implying the positive regulations of *CsRALF4/19* by *CsALC* is female specific. Considering the crossing data of male sterility and reduced female fertility in *Csralf4 Csralf19* double mutants (Figs. 5 and 6), CsRALF4/19 may have distinct functions in male and female cells in cucumber. Notably, the phenotype of seed set decrease in *Csralf4 Csralf19* double mutants was not as severe as that in *Csalc* mutants, indicating there are additional downstream genes of CsALC besides *CsRALF4/19* during pollen tube guidance (Fig. 7).

As the paralog of SPT, ALC originated from a gene duplication event prior to the emergence of the Brassicaceae^29^. In the dehiscent silique of *Arabidopsis*, *ALC* controls fruit opening by promoting cell differentiation at separation layers^27^. In the berry fruit of Solanaceae, transient knock-down of both *ALC* and *SPT* genes resulted in increased fruit lignification in tomato and pepper^45^. In the fleshy pepo fruit of Cucurbitaceae, our study showed the unique role of CsALC in female fertility via mediating pollen tube emergence in cucumber (Fig. 2, Fig. 7). Therefore, ALC has undergone functional divergence in different fruit types. From an evolutionary point of view, however, ALC homologs from different species commonly contribute to sexual reproduction success, such as participating in fruit opening and thus facilitating seed dispersal in *Arabidopsis*^46^, or controlling pollen tube guidance and enabling seed genesis in cucumber.

Interestingly, CsALC surrounding region is under-selected during the domestication process in cucumber (Supplementary Fig. 8). In wild cucumbers, seedy fruits are advantageous for species reproduction and survival. However, in cultivated cucumber, fruits with fewer seeds are preferred for easy consumption. Thus, the *CsALC* gene may be selected unconsciously during the long history of cucumber cultivation. Notably, a segment in the first exon of *CsALC* (not the bHLH domain) displayed no polymorphism among all the 115 cucumber germplasms examined, which encoded a serine that is highly conserved in the Cucurbitaceae family. The functions of this specific serine, as well as the key domestication regions of CsALC await further studies.

## Methods

### Plant materials and growth conditions

Cucumber (*Cucumis sativus* L.) inbred line XTMC (North China type) was used in this study. Seeds were soaked and germinated in the dark at 28 °C, then grown in the soil in a growth chamber under a 16 h/8 h and 28 °C/22 °C day/night regime until the roots fully developed and true leaves expanded. Seedlings were then transplanted to a standard greenhouse in the experimental field of China Agricultural University, Beijing. Water and fertilizer management, and pest control were performed according to standard protocols.

### Gene cloning and structural analysis

An 1140-bp fragment containing the complete *CsALC* coding sequence (CDS) was amplified from male flower buds and open male flowers, and the 351-bp length *CsRALF4* CDS and 339-bp length *CsRALF19* CDS were obtained in the same way with gene-specific primers (Supplementary Data 1), based on sequence information in CuGenDB (http://cucurbitgenomics.org/). The gene structure was analyzed using the online software GSDS 2.0 (http://gsds.gao-lab.org/).

### Phylogenetic trees construction

To retrieve protein and CDS sequences for phylogenetic analysis, *Arabidopsis* SPT and ALC were used as queries to search against the genome database Phytozome (https://phytozome.jgi.doe.gov/pz/portal.html) and National Center for Biotechnology Information (https://blast.ncbi.nlm.nih.gov/Blast.cgi), using BLAST with a parameter of 1 × 10^−10^. Full-length protein sequences of sampled species were aligned using the program Muscle 3.6 (http://www.drive5.com/muscle/), and the resulting alignment was manually adjusted in GeneDoc 3.2. Based on the protein alignment, a DNA matrix was produced by PAL2NAL (http://www.bork.embl.de/pal2nal/). Protein sequences of RALFs from *Arabidopsis* and cucumber were obtained using the protein BLAST search in NCBI, TAIR (https://www.Arabidopsis.org/), and CuGenDB. A phylogenetic tree was generated using the Neighbor-joining method and the bootstrap analysis with 1000 replicates in the MEGA-X. Multiple sequence alignment was performed using CLUSTALW in MEGA-X. Accession numbers of all sequences used for phylogenetic analysis are listed in Supplementary Data 2.

### Subcellular localization of CsALC

The full-length CDS of *CsALC* without termination codon was fused into the pUC-SPYNE vector to generate a CsALC-GFP in-frame fusion protein. The empty pUC-SPYNE vector was used as a positive control. Constructs were bombarded into onion epidermal cells using a PDS-1000/He particle delivery system (Bio-Rad) as described previously^47^. After incubation in the dark at 22 °C for 24 h, fluorescence signals were captured using a confocal laser-scanning microscope (Leica SP8, Germany) with 488 nm excitation wavelength.

### Quantitative real-time PCR

The young leaves, stems, tendrils, male flower buds at stage 10, female flower buds at stage 10, male flowers, female flowers, ovaries and pollens, pollen tubes at 3 h after germination, transmitting tract, ovules were frozen in the liquid nitrogen and stored at −80 °C until use. Unless otherwise indicated, these reproductive tissues and organs were collected at anthesis (the day for pollination). Transmitting tracts and ovules were isolated from the middle parts of the ovary. Pollens from four male flowers were eluted in RNA-extracted lysis buffer. Pollens were cultured in liquid pollen germination medium [15% (w/v) sucrose, 0.01% (w/v) boric acid, 1 mM Ca(NO_3_)_2_, pH 6.5] for 3 h and after centrifugal collection, pollen tubes were re-suspended in RNA-extracted lysis buffer. Total RNA was extracted with Trizol reagent as described in the manufacturer’s instructions (Promega, China, http://promega.bioon.com.cn/), and cDNA was synthesized using FastQuant RT Kit (Tiangen, China, http://www.tiangen.com/). Quantitative real-time PCR (qPCR) was performed using TB Green™ Premix Ex Taq™ (Takara, Japan, http://www.takarabiomed.com.cn/) on the Applied Biosystems 7500 real-time PCR system and Bio-RAD CFX384 system. Cucumber *Ubiquitin extension protein* (*CsUBI* CsaV3_5G031430) was used as the internal reference gene^48^. The relative expression was calculated according to the comparative cycle threshold (CT) method^49^. Three biological replicates and three technical replicates were performed for each gene. Primer information is listed in Supplementary Data 1.

### In situ hybridization

The 25-day-old shoot apex, male and female flower buds, and ovaries at anthesis without pollination and at 32 h after pollination of XTMC and *Csalc* mutants were fixed with 3.7% formalin–acetic acid–alcohol (FAA) fixative. Sampling and recognition of flower buds at different developmental stages were referred to in the previous study^50^. Sense and antisense probes were amplified with gene-specific primers containing SP6 and T7 RNA polymerase binding sites, respectively. Sample fixation, sectioning, and hybridization were performed as described previously^51^. Primer information is listed in Supplementary Data 1.

### Cucumber transformation

The CRISPR-Cas9 knockout vector pKSE402 with GFP fluorescence screening marker is provided by Prof. Huang Sanwen. The guide RNA (gRNA) is designed in the Cas-Designer website (http://www.rgenome.net/cas-designer/). Single target primers (for mutation of *CsALC*, *CsRALF4*) and double target primers (For simultaneous mutation of *CsRALF4* and *CsRALF19*) were designed and synthesized according to the published methods^52^. Single target gRNA generated by denaturing annealed, and double target gRNAs by PCR amplification using vector pCBC-DT1T2 as template were respectively incorporated into vector pKSE402. *Agrobacterium tumefaciens* EHA105 carrying the recombinant vector was shake-cultured to OD_600_ 0.6–0.8, then adjusted to OD_600_ 0.2–0.3. The cucumber cotyledon explant was infected by *Agrobacterium* suspensions with a syringe under negative pressure. After 3 days of co-culture in darkness, explants were transferred to the medium for bud differentiation and bacteriostasis. After 3–4 weeks, the regenerated shoots with GFP fluorescence were screened under a fluorescence stereomicroscope (Filter Cube: Excitation filters 460–500 nm, Dichroic mirror 505 nm, and Barrier filters 510 nm). The homozygous T1 mutant seedlings without vector (GFP-free) were selected from T0 transgenic lines for further phenotypic analysis and data statistics. Details about cucumber tissue culture refer to the previous study^53^.

### Alexander staining for pollen viability

Stamens from open male flowers, at least 3 for each plant, were washed to make pollen suspension (each flower stamen with 200 μL H_2_O). 5 μL pollen suspension and 15 μL Alexander dye were mixed on the slide and stained for 2 min. The pollen grains stained with purplish red under the microscope indicate the viable pollens. Each biological replicate counted at least 5 fields.

### in vitro pollen germination

Pollen grains from open male flowers were lightly swept into the pollen germination medium [15% (w/v) sucrose, 0.01% (w/v) boric acid, 1 mM Ca(NO_3_)_2_, and 0.5% (w/v) agar, pH 6.5] using a soft brush^54^. After being cultured in light and humid at 28 °C for 3 h, the germinating pollen tubes were observed and captured under microscope. The pollen tube germination percentage and pollen tube length were counted and measured in CellSens standard and ImageJ software^55^. At least three male flowers were counted for each mutant line and five fields were counted for each Petri dish. Pollen tube length longer than pollen grain diameter is determined as normal germination.

### Aniline blue staining of pollen tubes

One female flower was pollinated with two male flowers. Pollinated excised pistils (with superior ovaries) were placed in Petri dishes and wetly cultured in a growth chamber under a 16 h/8 h and 28 °C/22 °C day/night regime. After 32 h, the pistils were sliced lengthwise or crosswise (about 0.5–1 mm in thickness) and fixed in 75% ethanol and 25% acetic acid solution for 3–6 h. After softened in 8 M NaOH for 3–6 h, the sample slices were stained with Aniline blue solution [0.1% (w/v) Aniline Blue in 0.1 M K_3_PO_4_] for at least 2 h. The pistil slice was placed on a slide and the pollen tube extension in vivo was observed and photographed under a fluorescence microscope with excitation wavelength of 330–385 nm^56^. The pollen tube density in pistils was indicated by fluorescence intensity and quantitated in ImageJ software^55^. After fluorescence images were split into three channels, the red channel diagram was chosen and the fluorescence intensity was converted to the gray value, adjusted, and calculated in ImageJ software.

### Alcian blue staining of transmitting tracts

Pistils at anthesis were fixed in 3.7% formaldehyde, 5% acetic acid, and 50% ethanol and subsequently dehydrated through ethanol series (50%, 70%, 85%, 95%, 100%, each 60 min), then hyalinized with xylene (50% 60 min, 100% 60 min three times) and embedded in paraffin. Samples (styles and ovaries) in paraffin were made for 10 μm thickness sections by microtome. After deparaffinization through xylene (10 min twice) and ethanol series (100%, 95%, 85%, 70%, 50%, each 5 min), sections were hydrated in water twice and stained with 0.5% Alcian blue solution (1 g Alcian blue 8GX in 200 mL H_2_O) for 30 min. After washing, sections were examined under microscopy^56^. The deeper blue refers to the higher acid polysaccharide content.

### Transcriptomic analysis

Ovaries of WT and *Csalc* mutant at 32 h after pollination were collected for RNA-seq analysis. Three biological replicates were set. Illumina’s next-generation high-throughput sequencing platform and PE150 sequencing strategy were adopted. After quality control, clean read pairs were obtained and mapped to the cucumber genome sequence (http://cucurbitgenomics.org/ Chinese long CDS V2). All genes were quantitatively analyzed to identify the differential genes. The cutoff for DEGs was at least a 2-fold change in expression and a false discovery rate (FDR) of <0.05^57^ (listed in Supplementary Data 3). Sequencing data were deposited with the Gene Expression Omnibus (GEO) database at the National Center for Biotechnology Information under accession number GSE182139.

### Yeast one-hybrid assay

Vectors pB42AD and pLacZi2u were used for yeast one-hybrid assay. Plasmids for AD-fusions of CsALC were co-transformed with the LacZ reporters driven by two fragments from *CsRALF4* or 3 fragments from *CsRALF19* into the yeast strain EGY48. G-box elements were queried on *CsRALF4/19* promoter and genomic region, as well as other 27 DEGs between *Csalc* mutant and WT in the PlantCARE website (http://bioinformatics.psb.ugent.be/webtools/plantcare/html/). Yeast transformants were then selected on SD/-Trp-Ura agar plates for 4 days at 30 °C. The yeast transformants were further grown on SD/Gal/Raf/-Trp-Ura agar plates containing X-Gal (5-bromo-4-chloro-3-indolyl-β-d-galactopyranoside) for blue color development^58^. Yeast transformation was conducted as described in the Yeast Protocols Handbook (Clontech).

### LUC/REN assay for protein–DNA interactions in tobacco leaves

The *CsALC* CDS was cloned into pGreenII 62-SK as effectors. The 1811 and 3326 bp sequences upstream of *CsRALF4* and *CsRALF19* coding regions were cloned into pGreenII 0800-LUC as reporters. The *Agrobacterium* GV3101 pSoup strain carrying the above recombinant vector (9: 1 effector: reporter construct cell culture mixture) was introduced into the half of a *N. benthamiana* leaf. The other half leaf was infiltrated with the strain carrying the empty effector and *CsRALF4*/*CsRALF19* reporter as a negative control. The activities of firefly luciferase (LUC) and *Renilla reiformis* luciferase (REN) were measured at 72 h after infiltration on a microplate reader instrument (Molecular Devices SpectraMax i3x, the USA). The ratio of LUC to REN was calculated as the measurement result of transcriptional activity of CsALC on *CsRALF4*/*19* with eight biological replicates^59^.

### Nucleotide diversity and selective footprint analysis

Published sequencing data of 115 cucumber germplasms with 37 East Asian (EA), 29 Eurasian (EU), 19 Xishuangbanna (XSBN), 13 Indian wild genotypes (INW), and 17 Indian domestic (IND) accessions were used in this analysis^28^. Nucleotide diversity was calculated as an *π* value for the five groups using VCFtools, which was further used to estimate the fixation index (FST) between EA/EU/XSBN/IND and the Indian wild counterparts (INW).

### Reporting summary

Further information on research design is available in the Nature Portfolio Reporting Summary linked to this article.

## Supplementary information

Supplementary Information

Description of Additional Supplementary Files

Supplementary Data 1

Supplementary Data 2

Supplementary Data 3

Supplementary Data 4

Reporting Summary

## Data Availability

Transcriptomic data in this study can be found under accession number GSE182139 (https://www.ncbi.nlm.nih.gov/geo/query/acc.cgi?acc=GSE182139). Cucumber genes reported in this manuscript can be searched in CuGenDB (http://cucurbitgenomics.org/). All data generated or analyzed during this study are available in this published article and its supplementary information files. Source data are provided with this paper.

## Source data

Source Data
