# Supplementary material for: Pollen tube emergence is mediated by ovary-expressed ALCATRAZ in cucumber
Source: Nat Commun. 2023 Jan 17;14:258. doi: 10.1038/s41467-023-35936-z (PMC9845374; doi:10.1038/s41467-023-35936-z)
Supplement: Supplementary file 1 — Supplementary Information [file 41467_2023_35936_MOESM1_ESM.pdf]

Pollen tube emergence is mediated by ovary-expressed ALCATRAZ in cucumber

Zhihua Cheng[1#], Xiaofeng Liu[1#], Shuangshuang Yan[1], Bin Liu[2], Yanting Zhong[1], Weiyuan Song[1], Jiacai Chen[1], Zhongyi Wang[1], Gen Che[1], Liu Liu[1], Ao Ying[1], Hanli Lv[1], Lijie Han[1], Min Li[1], Jianyu Zhao[1], Junqiang Xu[3], Zhengan Yang[3], Zhaoyang Zhou[1*], and Xiaolan Zhang[1*]

[1]State Key Laboratories of Agrobiotechnology, Joint International Research Laboratory of Crop Molecular Breeding, Beijing Key Laboratory of Growth and Developmental Regulation for Protected Vegetable Crops, Department of Vegetable Sciences, China Agricultural University, Beijing 100193, China.

[2]Centre for Research in Agricultural Genomics (CRAG), CSIC-IRTA-UAB-UB, Campus Universitat Autònoma de Barcelona, Bellaterra 08193, Spain.

[3]College of Horticulture and Landscape, Yunnan Agricultural University, Kunming, Yunnan 650201, China.

[#]These authors contributed equally.

[*]**Corresponding authors:**

Xiaolan Zhang: zhxiaolan@cau.edu.cn; Tel: (86)10-62732102

Zhaoyang Zhou: zyzhou@cau.edu.cn; Tel: (86)10-62732702

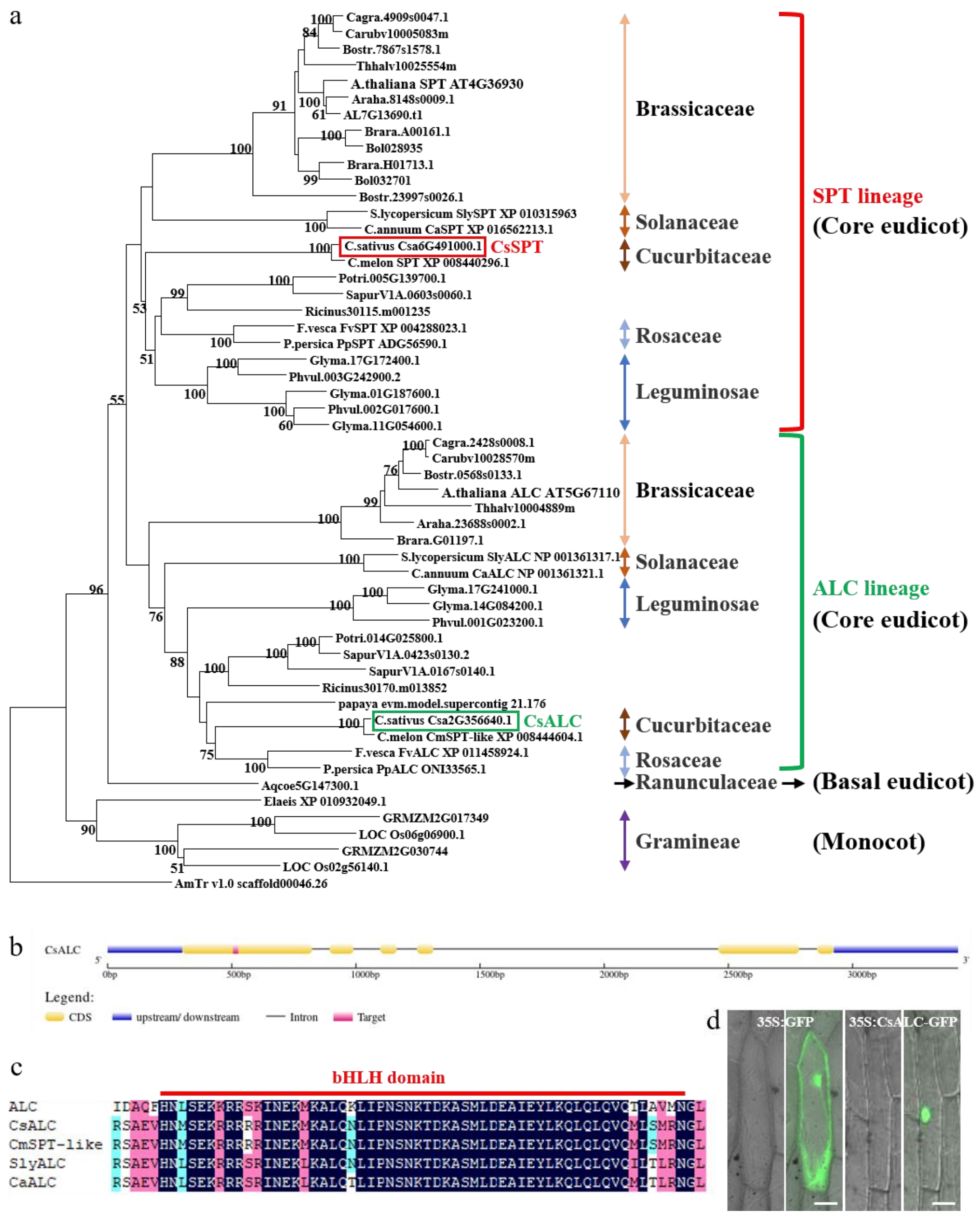

Supplementary Figure 1. Sequence analyses for CsALC. a Phylogenetic analysis of CsSPT and CsALC homologues among angiosperms. **b** *CsALC* gene structure, in which exons and introns are marked by yellow boxes and black lines, respectively; pink box indicates the gRNA-targeted 19nt region; blue boxes represent the 5’ and 3’UTRs. **c** bHLH domain alignment of CsALC and representative ALC homologues selected from (**a**). **d** Subcellular localization of CsALC. GFP driven by the 35S promoter was used as the positive control. GFP was shown in green. Left and right represent pictures taken under bright field and merge views, respectively. The experiment was repeated twice with similar results. Scale bars = 50 μm.

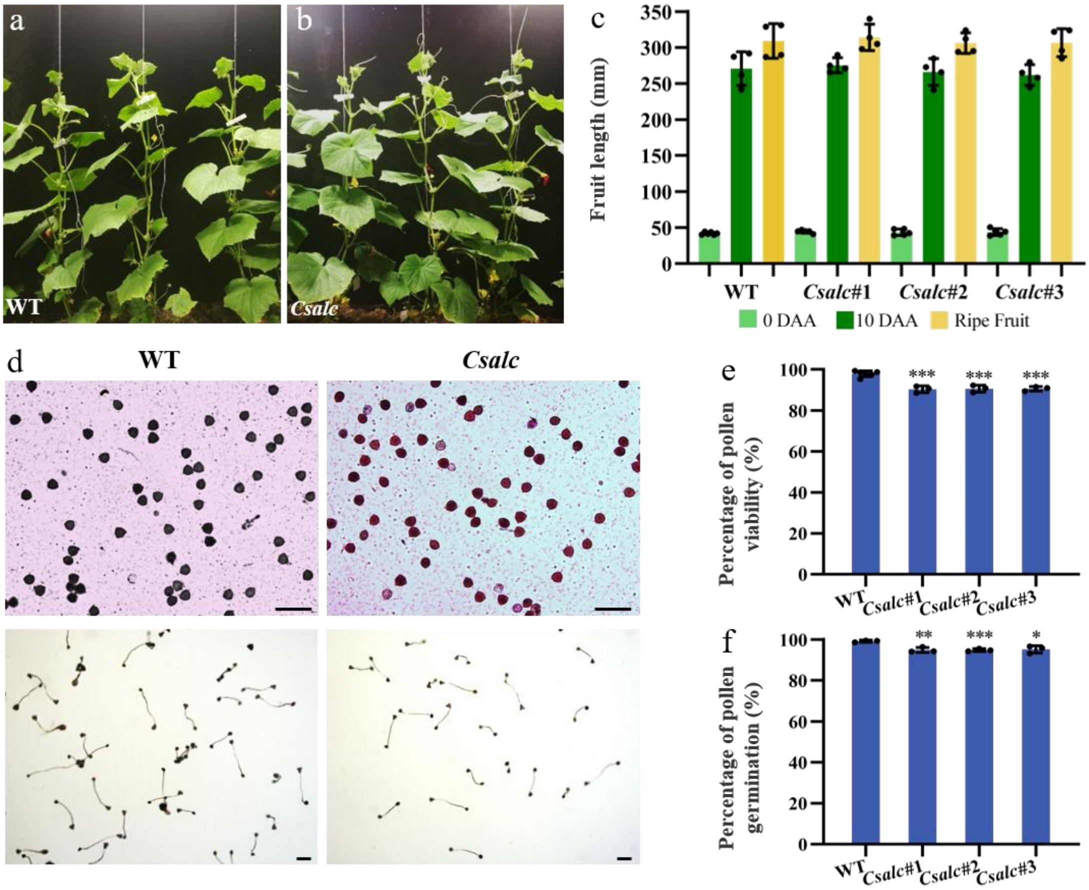

Supplementary Figure 2 Comparison of WT and *Csalc* mutants in plant growth, fruit length and pollen viability. a-b Plant growth of WT and *Csalc* mutants in the greenhouse. **c** Quantitative analysis of WT and *Csalc* fruit length at three developmental stages. DAA: days after anthesis. From left to right, n = 6, 4, 4, 5, 4, 4, 5, 4, 4, 5, 4, 4 ovaries. **d**, Alexander staining of pollens (top row) and *in vitro* pollen germination (bottom row) in WT and *Csalc* mutants. Scale bars = 200 µm. **e**, **f** Quantitative analysis of pollen viability in (**d**). n = 6, 3, 3, 3 biologically independent samples in (**e**) and 3, 3, 3, 3 biologically independent samples in (**f**), respectively. *$p < 0.05$, ** $p < 0.01$, *** $p < 0.001$ (two-sided Student's t test).

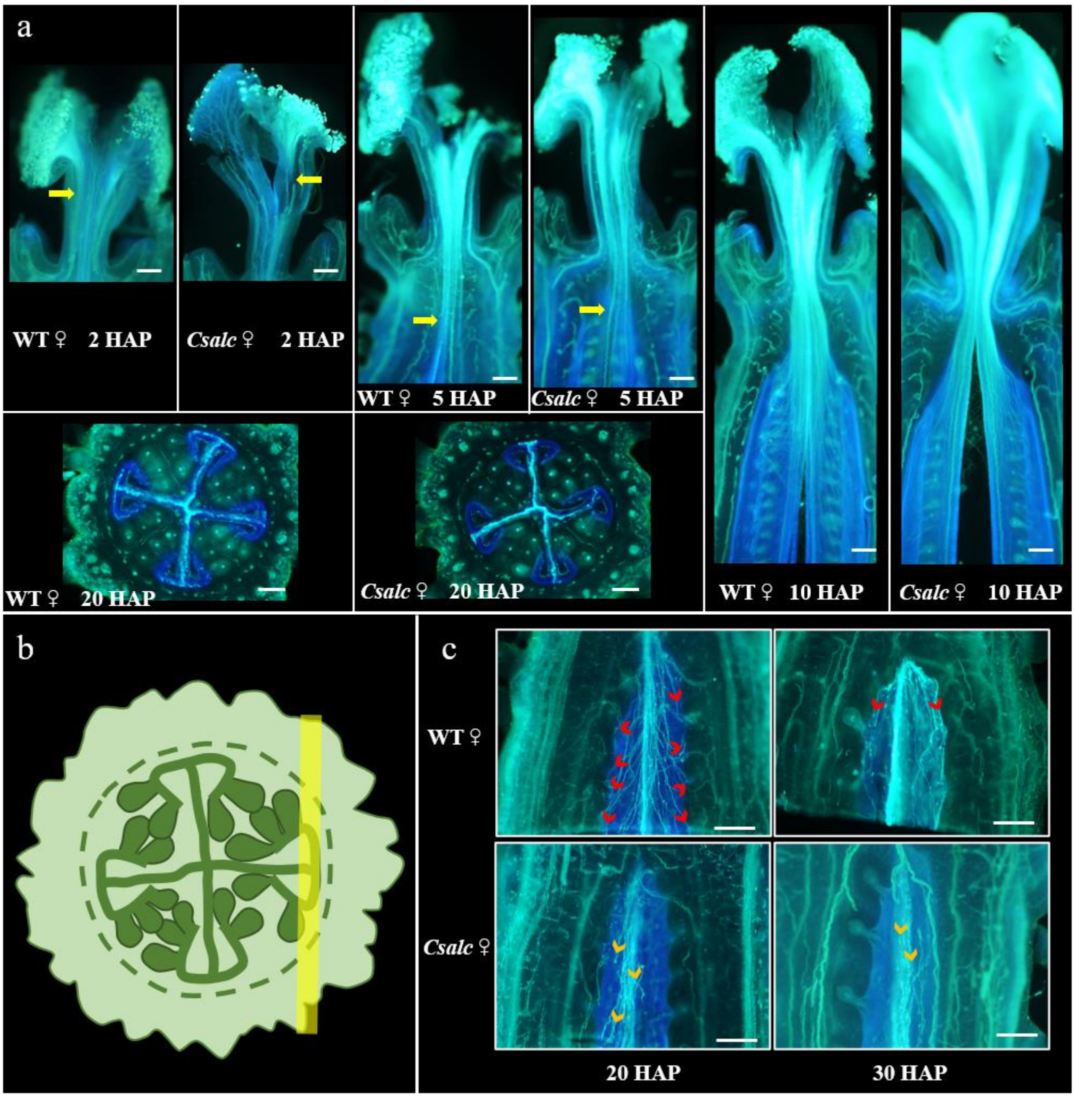

Supplementary Figure 3 Comparison of pollen tube extension in WT and *Csalc* mutants. a Pollen tube extension in WT and *Csalc* pistils at 2 h, 5 h, 10 h, 20 h after pollination (HAP). Yellow arrows indicate the pistil position where most pollen tubes reached. Composite images were integrated and spliced from local images taken separately. Scale bars = 500 μm. **b-c** Pollen tube extension at lateral TT in WT and *Csalc* mutants. (**b**) Schematic diagram of ovary transection with four carpels. The yellow strip refers to the position of longitudinal sections in (**c**). **c** Pollen tube distribution at lateral TT of WT and *Csalc* mutants at 20 HAP and 30 HAP. Red/ Orange arrow heads indicate the WT pollen tube extension direction in WT/ *Csalc* mutants. Scale bars = 500 μm. The experiments were repeated three times with similar results (**a, c**).

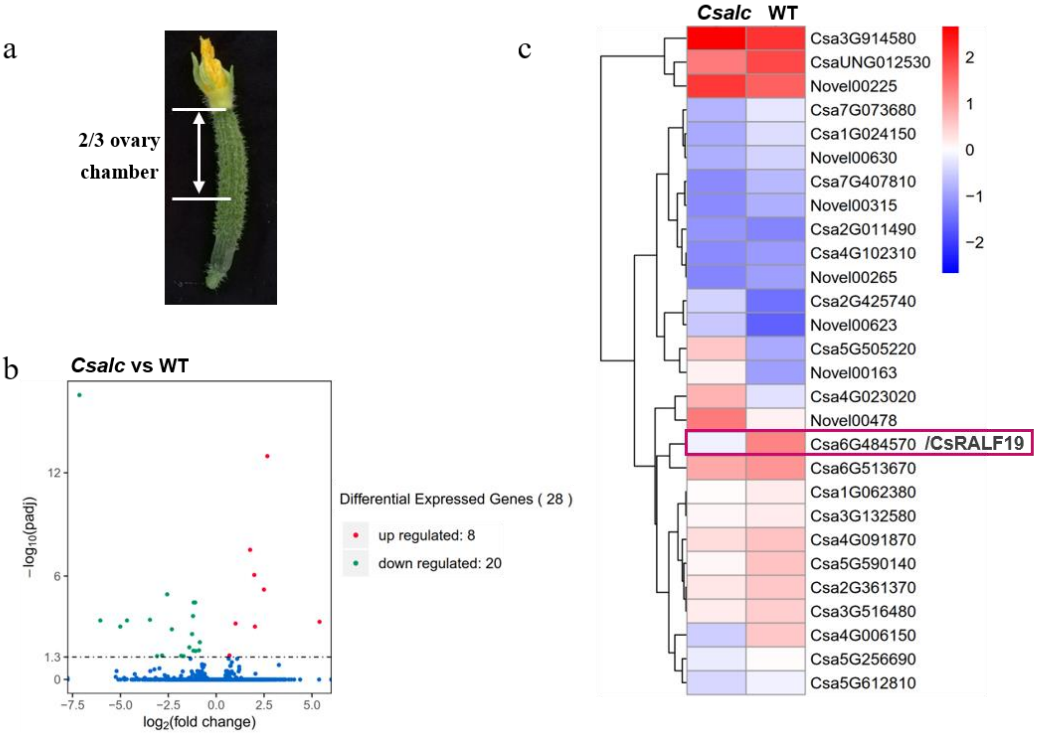

d

	readcount_*Csalc*	readcount_WT	log2FoldChange	pval	padj
CsRALF19	55.31	607.90	-3.46	1.61E-07	3.49E-04

Supplementary Figure 4 Transcriptomic analyses of *Csalc* mutant and WT ovaries. **a** A representative ovary sample (top 2/3) at 32 h after pollination used for RNA-Seq. **b** Volcano of DEGs between *Csalc* mutant and WT. **c** Heat map of the 28 DEGs between *Csalc* mutant and WT. **d** Quantitative data of *CsRALF19* expression in *Csalc* mutant and WT ovaries by RNA-Seq.

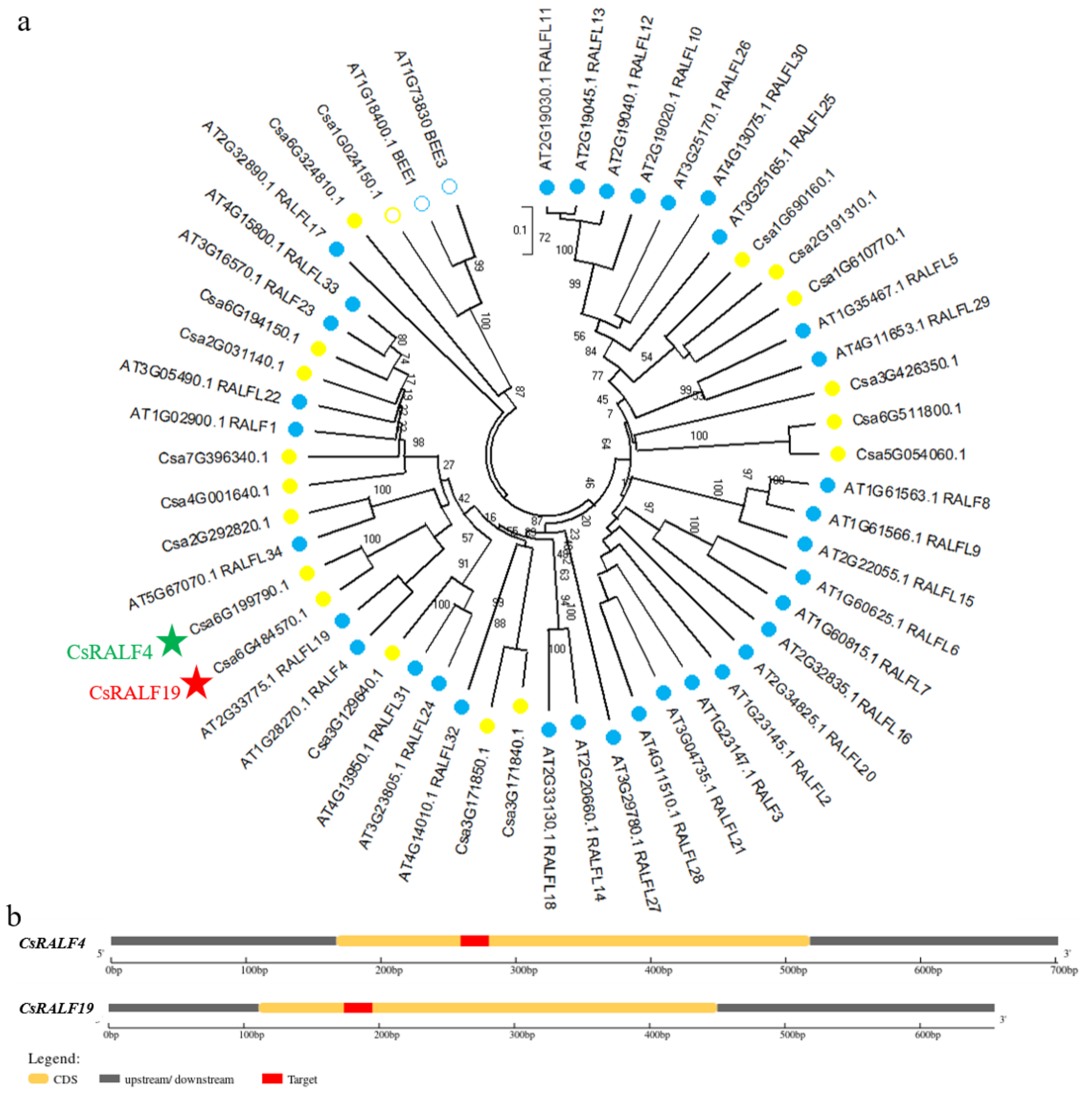

Supplementary Figure 5 Phylogenetic and sequence analyses for *CsRALF4* and *CsRALF19* gene. a Cucumber RALF4/19 clustered with *Arabidopsis* homologues. Blue solid circles, *Arabidopsis* RALFs; Yellow solid circles, potential cucumber RALFs. *Arabidopsis* and cucumber bHLH proteins (blue and yellow hollow circles) were used as the outgroup. **b** Structural diagram of *CsRALF4* and *CsRALF19* genes. Exons are marked by orange boxes, red box indicates the gRNA-targeted 19nt region, and gray boxes represent the 5' and 3'UTRs.

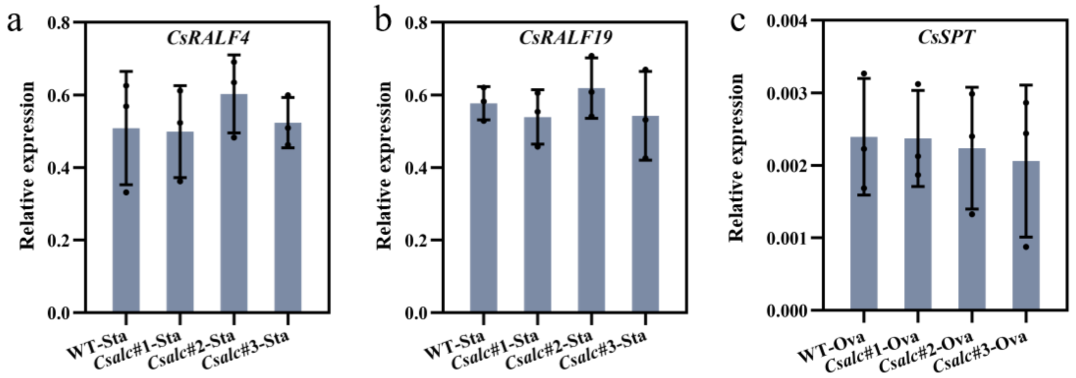

Supplementary Figure 6 qPCR analysis of *CsRALF4/19* and *CsSPT* in *Csalc* mutants. a-b *CsRALF4* (**a**) and *CsRALF19* (**b**) expression in WT and *Csalc* stamens. **c** *CsSPT* expression in WT and *Csalc* ovaries. n = 3 biologically independent samples. Error bars represent mean + SD.

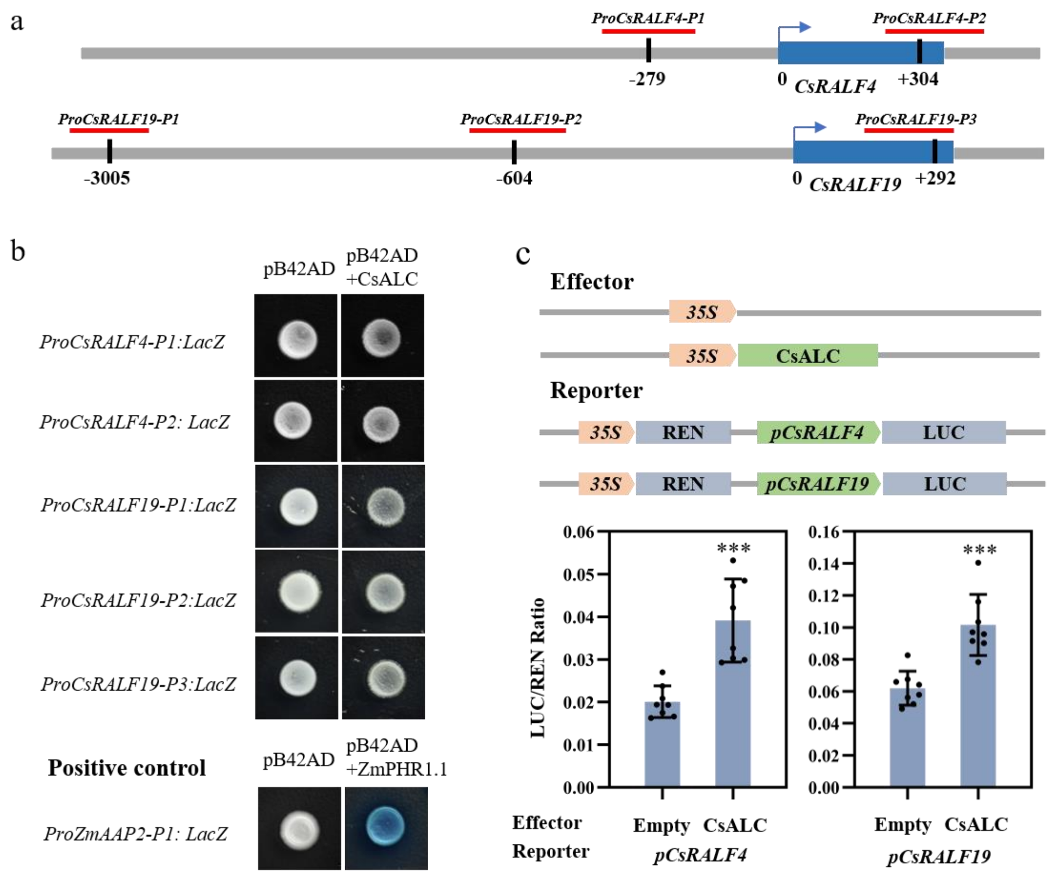

Supplementary Figure 7 CsALC promoted *CsRALF4/19* expression *in vivo*. a Schematic diagram of fragments containing G-box elements (*ProCsRALF4-P1*/*P2*; *ProCsRALF19-P1*/*P2*/*P3*) in promoters and genomic regions of *CsRALF4* and *CsRALF19*, respectively. **b** Yeast one hybrid assay showing no binding of CsALC to segments containing the G-box elements in *CsRALF4* or *CsRALF19* promoter and genomic region. The protein ZmPHR1.1 and the segment *ProZmAAP2-P1* was used as the positive control[58]. **c** LUC activity assay in *Nicotiana benthamiana* leaves after co-expression of 35S:CsALC (effector) and *pCsRALF4:LUC*/*pCsRALF19:LUC* (reporter). The empty vector effector was used as the negative control. The REN gene driven by the 35S promoter was used as the internal reference. n = 8 biologically independent samples. Error bars represent mean + SD. *** $p < 0.001$ (two-sided Student's *t* test).

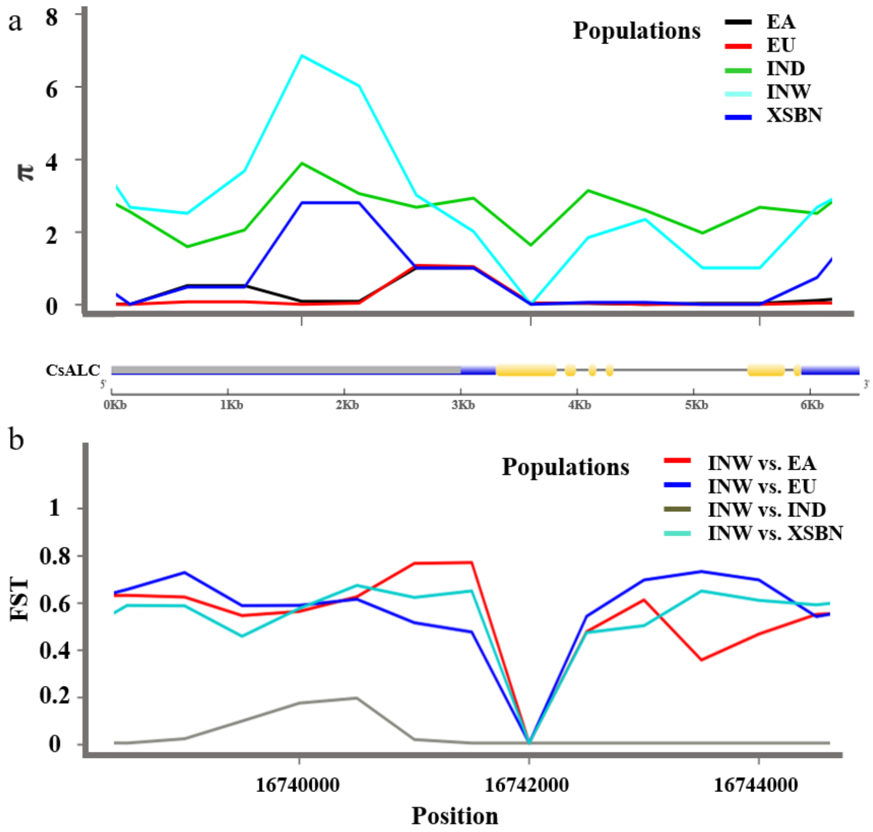

Supplementary Figure 8 The region surrounding CsALC gene was selected during cucumber domestication. a Nucleotide diversity of five cucumber populations at individual nucleotide sites in CsALC gene region. The horizontal axis refers to the position in the Chinese Long 9930 v2 genome, and the vertical axis represents the π value of each cucumber group. **b** Pairwise difference of allele frequency (FST). The horizontal axis indicates the position in Chinese Long 9930 v2 genome, and the vertical axis refers to FST value of INW vs. EA, INW vs. EU, INW vs. IND, and INW vs. XSBN. INW: Indian wild, IND: Indian domestic, XSBN: Xishuangbanna, EA: East Asian, EU: Eurasian.

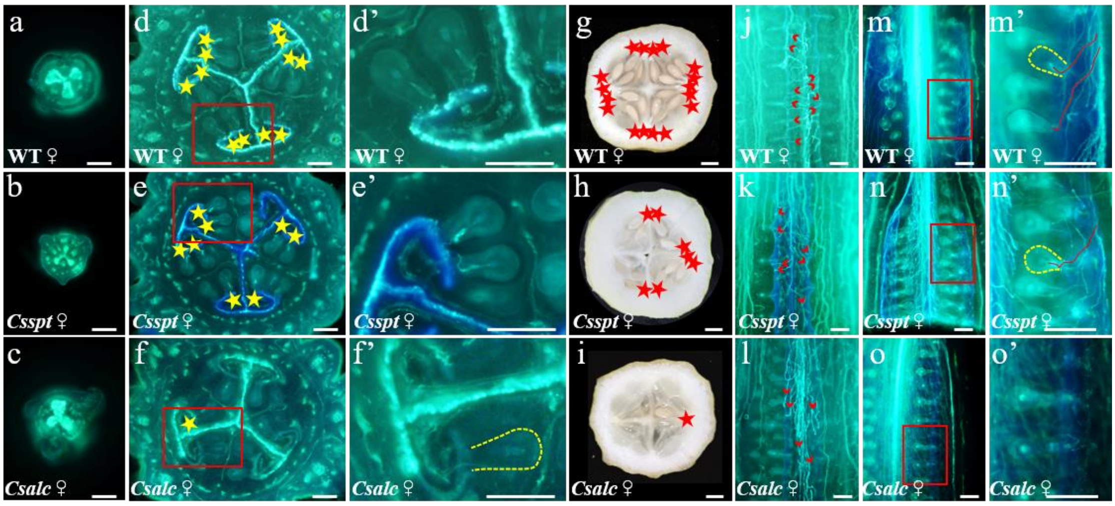

Supplementary Figure 9 The comparison of pollen tube extension in *Csspt* and *Csalc* mutants. a-f' *Csspt* mutant displaying extremely reduced pollen tube density while relatively normal ovule targeting compared to *Csalc* mutant. (**a-c**) Transverse sections of styles; (**d-f**) Top quarter of ovaries; (**d'-f'**) Enlarged view of red boxes in (**d-f**). Yellow stars mark ovules targeted by pollen tubes. Scale bars = 500 µm. **g-i** Transection of WT, *Csspt* and *Csalc* mature fruits. Red stars indicate the plump seeds. Scale bars = 1 cm. **j-l** Normal pollen tube emergence at lateral TT in *Csspt* mutant. Red arrow heads refer to the pollen tube extension direction. Scale bars = 500 µm. **m-o**' Longitudinal sections showing WT pollen tubes enter ovules in WT and *Csspt* mutants. **m'-o'** Enlarged view of red boxes in (**m-o**). Targeted ovules are delineated by yellow dots, and pollen tubes are marked by red lines. Scale bars = 500 µm. The experiments were repeated three times with similar results.
