# Supplementary material for: Pollen tube emergence is mediated by ovary-expressed ALCATRAZ in cucumber
Source: Nat Commun. 2023 Jan 17;14:258. doi: 10.1038/s41467-023-35936-z (PMC9845374; doi:10.1038/s41467-023-35936-z)
Supplement: Supplementary file 2 — Description of Additional Supplementary Files [file 41467_2023_35936_MOESM2_ESM.pdf]

Description of Additional Supplementary Files:

Supplementary Data 1: Primer information.

Supplementary Data 2: SPT and ALC homologues among angiosperms.

Supplementary Data 3: DEGs between Csalc mutants and WT.

Supplementary Data 4: G-box distribution in Csalc DEG promoters
